# Prevention and postvention guidance relating to self-harm and suicide for UK educational and youth organisations: a systematic review of grey literature

**DOI:** 10.1186/s12889-026-27052-6

**Published:** 2026-03-18

**Authors:** Emily Widnall, Hannah Robinson, Lingyi Wang, Gregory Hartwell, Rowan Streckfuss Davis, Frances Mathews, Faraz Mughal, Liam Spencer, Sally O’Keeffe, Abigail Russell, Lucy Biddle, Judi Kidger

**Affiliations:** 1 Population Health Sciences, Bristol Medical School, Canynge Hall, University of Bristol, Bristol, BS8 2PL UK; 2 London School of Hygiene and Tropical Medicine, London, UK; 3 University of Exeter, Exete, UK; 4 Oxford Primary Care Clinical Trials Unit, Nuffield Department of Primary Care Health Sciences, University of Oxford, Oxford, UK; 5 Newcastle University, Newcastle, UK

**Keywords:** Self-harm, Suicide prevention, Schools, Youth organisations, Guidance, Education, Systematic review

## Abstract

**Introduction:**

Self-harm and suicide among young people are major public health concerns and delivering prevention and postvention support is crucial. Educational and youth organisations play a vital role in both prevention and postvention. However, evidence shows a lack of understanding of effective interventions, and limited awareness of the available guidance in this area. We reviewed current self-harm and suicide guidance available to education and youth organisations in the UK that support young people and identified gaps in this provision.

**Methods:**

A systematic grey literature review was conducted between July and August 2023 using the following methods: internet searches (Google and OpenGrey), advice from experts and reference list checks. To be included, guidance had to focus on supporting children and young people aged 11–18 years and be intended for use in education or youth organisation settings in the UK. Guidance also had to include information on self-harm and/or suicide prevention or postvention and be freely and publicly available. A narrative review and content analysis of all included guidance was conducted. Gaps in existing guidance were identified from the content analysis, through consultation with young people and adult advisory group members and comparison with relevant literature. A formal quality appraisal was not undertaken due to a lack of validated frameworks to assess grey literature.

**Results:**

Two hundred ninety-seven guidance resources were screened; 104 met the inclusion criteria. Regarding prevention guidance, 36 focused on self-harm, 4 on suicide and 21 covered both. By comparison, 29 covered postvention and 14 covered both prevention and postvention (8 were suicide only and six were self-harm and suicide). Notably, there was a marked imbalance in target settings, with over half of the guidance (54/104) focused on educational settings compared with only 5 resources specifically tailored to youth organisations, highlighting a substantial gap in guidance for non-education settings. Three key categories of content were identified: psychoeducation/information, recommended response and resources. Within these, 20 subcategories were identified: definitions and signposting were the most frequently included. The stakeholder-informed gap analysis revealed a lack of support for parents/carers, practical resources for professionals to use and tailored information for specific populations (e.g. neurodiverse, LGBTQIA+). Although 104 relevant guidance resources were available, it was difficult to assess the quality of the guidance as there was a lack of information on how individual guidance resources were developed and whether the content was evidence-based.

**Conclusion:**

Key recommendations for future guidance include more guidance for youth organisations, more on response and support after a suicide, more tailored guidance for vulnerable groups and more guidance for how organisations can support parents and carers. Additionally, guidance should be co-produced to ensure that key topics identified as important to stakeholders are not missed.

**Supplementary Information:**

The online version contains supplementary material available at 10.1186/s12889-026-27052-6.

## Introduction

Self-harm and suicide (SH&S) among young people are major public health concerns [1]. In 2023, 219 young people aged 10–19 years were registered to have died by suicide in England and Wales [2]. It is estimated that 7% of UK children have attempted suicide by age 17 and that the prevalence of ever having self-harmed (a strong precursor to suicide, although it should be noted that the absolute risk of suicide remains rare) at 17 is 24% [3]. Such deaths can create detrimental long-term impacts on the mental health of friends, peers, family members, and all those within the same social network. Importantly, this can extend to further suicidal behaviour – contagion (the spread of suicidal behaviours within a community or group) and clustering (a series of suicides or suicide attempts occurring closely in time or location) [4]. The youth population at risk of self-harm, suicidal behaviour, or of experiencing a bereavement by suicide is therefore substantial, and in need of both prevention and postvention (response following a death by suicide) support to reduce self-harm and suicidal behaviour, deliver bereavement care, and to promote recovery after experiencing a suicide of someone close.

Given the potentially traumatic impact of suicidal behaviour across a whole community [5], it is important to consider universal, organisational-level self-harm and suicide prevention and postvention approaches. Although schools, colleges, and youth organisations are well-placed to identify and intervene with those at-risk [6, 7], there remains limited evidence for effective school-based prevention, assessment, and support [8]. A recent qualitative study of the Samaritans’ suicide postvention service (‘Step by Step’) delivered to schools [9], revealed a lack of clarity about what prevention and postvention support exists, with most schools having no plan in place for how best to respond to a suicide. A UK study also found that self-harm is often invisible within educational settings and not prioritised within the health curriculum, and that an ‘escalation approach’ in which teaching staff inform senior management who then refer the young person to external experts could contribute to non-help seeking behaviour within schools [10].

Although a need for SH&S prevention and suicide postvention among youth populations is well-recognised in the scientific literature, policy documents, and by teachers [11–13], there is a lack of evidence about the characteristics of efficacious, useful, and helpful guidance or interventions. A recent UK systematic review on education and training interventions and support tools for school staff to respond to young people disclosing self-harm, found only eight studies [14]. Although all eight studies found increased knowledge, skills, and confidence of staff responding to self-harming youth, the review concluded that more evidence is needed to determine the effectiveness, acceptability, and feasibility of these types of interventions/tools [15]. A recent review of suicide prevention interventions in educational settings found no evidence of iatrogenic effects [16]. However, one case study of postvention in a school setting found that inappropriately delivered postvention can be harmful, potentially leading to imitative behaviour, and thus increasing suicide risk among students [5]. Indeed, those who work with young people - such as school staff - can be reluctant to openly talk about SH&S due to the fear of encouraging other young people to self-harm, despite young people discussing SH&S across a range of formats and spaces including within friendship groups, across social media spaces and whilst gaming [17]. Even less is known about the availability and effectiveness of self-harm or suicide interventions in other youth settings (e.g. sports clubs, faith-based groups Scouts/Girlguiding or other youth centres/clubs/organisations), with no published studies in these settings This gap persists despite the UK Government’s five-year suicide prevention strategy explicitly stating that suicide prevention is “the responsibility of multiple government departments, as well as wider public, private and VCSE sector organisations” [18].

An Australian Delphi study in 2016 developed suicide postvention guidelines for secondary schools [19]. This study searched for existing postvention guidelines developed for English-speaking secondary schools and designed an online questionnaire based on potential actions that school staff could carry out following the suicide of a student. This questionnaire was sent to 40 experts in suicide postvention who rated the actions in terms of how important they felt it was to be included in future guidelines. Over 500 actions were endorsed, centring around common themes including emergency response, management, liaising with family, informing students, parents and the wider community, identifying and supporting high risk students, dealing with the media, internet and social media, funeral and memorial, continued monitoring and future prevention. Experts often noted that every suicide was unique and therefore there is a need for flexibility in guidelines. A more recent US study published in 2023 explored both effectiveness data from postvention interventions as well as guidance recommendations from experts [20]. This study highlighted the importance of supporting students and staff through the grieving process as well as the need to involve mental health professionals, particularly for those who are at higher risk. One prevention Delphi study was conducted in New Zealand which aimed to develop guidelines for school staff to support students who self-harm, the study endorsed communication, collaborative responsibility, well-being and a student-centred approach [21]. Although a few guidance development studies exist in Australasia and the US, empirical research on UK guidance for self-harm and suicide prevention and postvention is currently lacking.

Two recently published reviews led by Australian authors have further synthesised the international postvention evidence-base. A systematic review explored the evidence for preparing for and responding to student suicide in school settings [22]. The review identified 19 school interventions (10 aimed at students and 9 aimed at staff) and found some evidence of benefit for students and staff however it noted poor quality of the studies and significant remaining knowledge gaps. Secondly, a scoping review of the effectiveness of postvention service models and guidelines revealed the promise of interventions such as psychoeducation, trained peers and workplace programmes [23]. However, this review was not focused on children and young people and included only two Italian school-based studies, again highlighting a gap in youth postvention responses, particularly within the UK context.

In the UK setting, there is a lack of awareness among professionals working with young people, and the organisations supporting these professionals, about the best ways in which to prevent self-harm and suicide, and support those who have self-harmed or been bereaved by a suicide. A GW4 alliance report found that schools were apprehensive to explicitly discuss self-harm with the student population due to fears of contagion and had reservations around universal self-harm interventions unless they were focused on mental health more broadly [24]. Furthermore, previous literature highlights a lack of awareness of what prevention and postvention support is available to them or their organisation and a lack of clarity and evidence regarding what the characteristics of efficacious guidance are [9].

Therefore, there is a need for further understanding around what UK self-harm and suicide guidance is currently available to education and youth organisations supporting young people, and identification of any gaps in what is available. Clear, contextually relevant guidance is critical for supporting effective implementation and staff training in educational and youth settings, particularly in relation to sensitive and high-risk issues such as self-harm and suicide. Without a comprehensive picture of what guidance exists, organisations may experience variability in training, uncertainty among staff and inconsistent responses to prevention and postvention situations, underscoring the importance of systematically identifying and analysing the content of guidance available within the UK context. At the same time, the mere availability of guidance does not guarantee that its recommendations are evidence-based or that their implementation leads to improved outcomes, further reinforcing the need to examine both its content and development.

This review focused on publicly available grey literature to answer the following research questions:

1. What guidance or resources are freely available in the UK that provide education and youth organisations with:
  i. prevention support for self-harm and suicide.
  ii. postvention support following a death by suicide
2. What are the gaps in current UK-based self-harm and suicide prevention and postvention guidance for education and youth organisations?

In this review, *prevention* refers to guidance aimed at reducing the risk of self-harm or suicide, including support for individuals engaging in current self-harm, while *postvention* refers to guidance addressing responses following a death by suicide.

## Methods

A systematic review of grey literature was conducted. For this review, the term ‘grey literature’ is defined as publicly available, open-source information, which is not controlled by commercial publishers [25]. A review protocol was developed in advance and registered on Open Science Framework (https://osf.io/tj9x6). A PRISMA checklist can be found in Supplementary Materials 1.

The searches focused exclusively on grey literature because prevention and postvention guidance for self-harm and suicide in UK educational and youth settings is most commonly produced outside of academic publishing. In the UK, such guidance is typically developed by government departments, local authorities, charities, professional bodies and third-sector organisations, and disseminated as reports, toolkits or policy documents rather than peer-reviewed journal articles. Restricting the review to grey literature therefore allowed for a more comprehensive and practice-relevant synthesis of the guidance currently available to schools and youth organisations, which would not be adequately captured through searches of published academic literature alone. In addition, several systematic reviews already synthesise the international peer-reviewed evidence base on suicide and self-harm prevention and postvention in educational settings; the present review was intended to complement this literature by focusing specifically on UK guidance used in practice.

Three methods were used to search for relevant guidance and resources: (1) internet searches; (2) advice from experts; and (3) reference list checks.

1. Internet searches
  Two separate searches were conducted in both Google (advanced search) and the OpenGrey database:
  - Self-harm and suicide prevention guidance
  - Suicide postvention guidance
    Searches took place between July and August 2023. A full copy of the search terms can be found in Supplementary Materials 2. The searches were restricted to English-language and UK-specific guidance, to ensure relevance to the UK context. To be included in the review, guidance had to have been updated within the past 10 years. The first 10 pages of Google records were screened, and a decision was made by the primary reviewer (EW) for when to stop screening beyond this as records were becoming less relevant. Relevance decline was judged pragmatically, with screening discontinued when consecutive pages predominantly yielded duplicate records, non-UK materials, non-guidance documents (e.g. news articles or opinion pieces), or resources unrelated to self-harm or suicide prevention and postvention in education or youth settings.
2. Advice from experts
  The authors gathered suggestions from experts and academics working in the field. Guidance documents suggested via experts were accepted between July 2023 and January 2024. Experts, academics and our young person advisors were identified through networks of the authors and contacted directly to suggest relevant organisations, websites or guidance. Additionally, third sector organisations such as key national and local prevention and postvention charities (e.g. Pete’s Dragons, Samaritans, Papyrus) were contacted to identify relevant guidance. This search strategy has been successful in identifying relevant grey literature in previous reviews [26, 27].
3. Reference lists
  Reference lists from included relevant resources identified in steps 1 and 2 were screened for additional resources by title, contents page and source. Those deemed relevant were read in full to assess eligibility for inclusion. 

### Eligibility criteria

The following inclusion and exclusion criteria were applied: 

#### Inclusion criteria

- Guidance is aimed at supporting children and young people aged 11–18 years.
- Guidance is for education or youth organisations in the United Kingdom.
- Guidance includes information for education or youth organisations on either self-harm and/or suicide prevention or suicide postvention.
- Guidance must be written in a webpage, report or PDF format.
- Guidance must be freely and publicly available.

#### Exclusion criteria

- Individual school/college self-harm or suicide protocols or policies.
- Guidance based on clinical populations.
- Guidance not tailored to youth populations (e.g. general NHS/NICE recommendations).
- Guidance for organisations that have no branch in the UK.
- Guidance for higher education (University) settings given that universities are predominantly young adult populations, therefore guidance for this setting may be less relevant for our age range.

For guidance that spanned multiple age groups or settings, inclusion was determined based on whether the content explicitly addressed children and young people aged 11–18 years or included guidance relevant to education or youth organisations; if the guidance only partially addressed this population or setting, it was included but flagged as multi-age or multi-setting for analysis purposes.

### Data management

The number of resources identified, screened, assessed for eligibility (initial screening and full text screening), excluded and included for review were stored in an Excel file along with the screening dates and a record of URL and/or PDF links to the guidance.

### Initial searches and screening

Searches and first screening were conducted by the primary reviewer (EW). Resources were screened based on their title and contents page (where applicable) and kept for full text screening if they met all the inclusion criteria listed above. Any resources that we were unable to exclude based on the title and contents were included for the full-text review. As part of this initial screening phase, a second reviewer (LW) double screened 15% of the full-text resources resulting in a 77% agreement rate between EW and LW. Discrepancies were discussed between the two reviewers to reach a decision and with the wider author team where necessary. The lead researcher is specifically experienced in school-based mental health research, which may have influenced decisions regarding relevance, categorisation, and interpretation of guidance content; reflexive discussions were therefore held throughout the review process to enhance transparency and minimise potential bias.

### Data extraction

An Excel data extraction form was created by the primary reviewer (EW) and data extraction was completed by three reviewers (EW, LW and NB). All extractions were checked and finalised by the lead reviewer (EW). The data extraction template captured (where these things were stated): Title of guidance, guidance URL, publishing organisation(s), date of publication and renewal date, target audience (e.g. type of organisation the guidance was written for), age range of young people, guidance focus (self-harm/suicide or both and prevention/postvention or both), overview of guidance contents, organisations/charities referred to/signposted to, any listed interventions and key references to screen.

### Data synthesis and reporting

We conducted a narrative review and content analysis of all included guidance [28]. Content analysis is an approach that combines qualitative and quantitative methods and allows researchers to classify sections of text into content-related categories and summarize content. In this case, the categories identified were labelled as ‘features’ of the guidance reviewed. These features were subsequently organised into key sections of content [29]. We constructed a visual summary of the prevalence of the ‘key features’ identified in the included guidance resources (Table 1). The review focused on mapping the scope and key features of available guidance, rather than evaluating the quality, accuracy or evidential validity of its content.

### Gap analysis

To support the primary content analysis, we conducted a supplementary gap analysis to help identify gaps within current guidance features, areas for improvement and considerations for future guidance provision. Gap analyses are used to identify inconsistencies between provider (guidance) and client (schools and youth organisations and young people) perceptions. The traditional gap analysis approach was developed by Parasuraman et al. (1985) as a service quality measurement tool which focused on the analysis of the gaps in perception between the service that consumers expected compared to what they actually received [30]. This gap analysis model has been used within health service improvement [31], but applying this method to a public health review of literature is an innovative application of this approach.

Two advisory groups were involved in the gap analysis; one made up of young people (YPAG) and one made up of adult professional stakeholders. The young person’s advisory group members were recruited from a local mental health charity (McPin) and the professionals were recruited through contacts with the study team, schools and local charities. Six young people (aged 21 and under) with lived experience of self-harm and/or suicidal thoughts were involved in the rating and gap analysis activity. Four adults who worked in roles relevant to self-harm and suicide in young people e.g., a suicide prevention charity, took part in the same activity.

In order to gain feedback from our two advisory groups, we constructed a visual summary of the features identified and their frequency in existing guidance. We then presented this visual to both advisory groups (details below) and explained what was meant by each feature, using examples from included guidance. We then asked group members to rate each feature in terms of importance. If a feature appeared with low frequency in the guidance but was rated as important, this was considered a gap. We also asked our advisory groups to identify anything they felt was important that was missing from all guidance.

### Ratings of importance and gap analysis activity

We asked members of our stakeholder advisory groups to individually rate on a scale of 1 to 5 (5 being very important, 1 being not important) each feature identified by the content analysis in terms of how important they thought it was that guidance includes this feature. All advisory group members also commented on the features and suggested potential areas of improvement and missing features as part of the gap analysis as described above. Qualitative comments from both advisory groups were integrated with the numerical importance ratings by reviewing suggestions alongside low-frequency features; if multiple participants highlighted a feature as missing or in need of improvement, this reinforced its identification as a gap, ensuring that both quantitative ratings and qualitative insights informed final gap decisions.

## Results

**Fig. 1:**
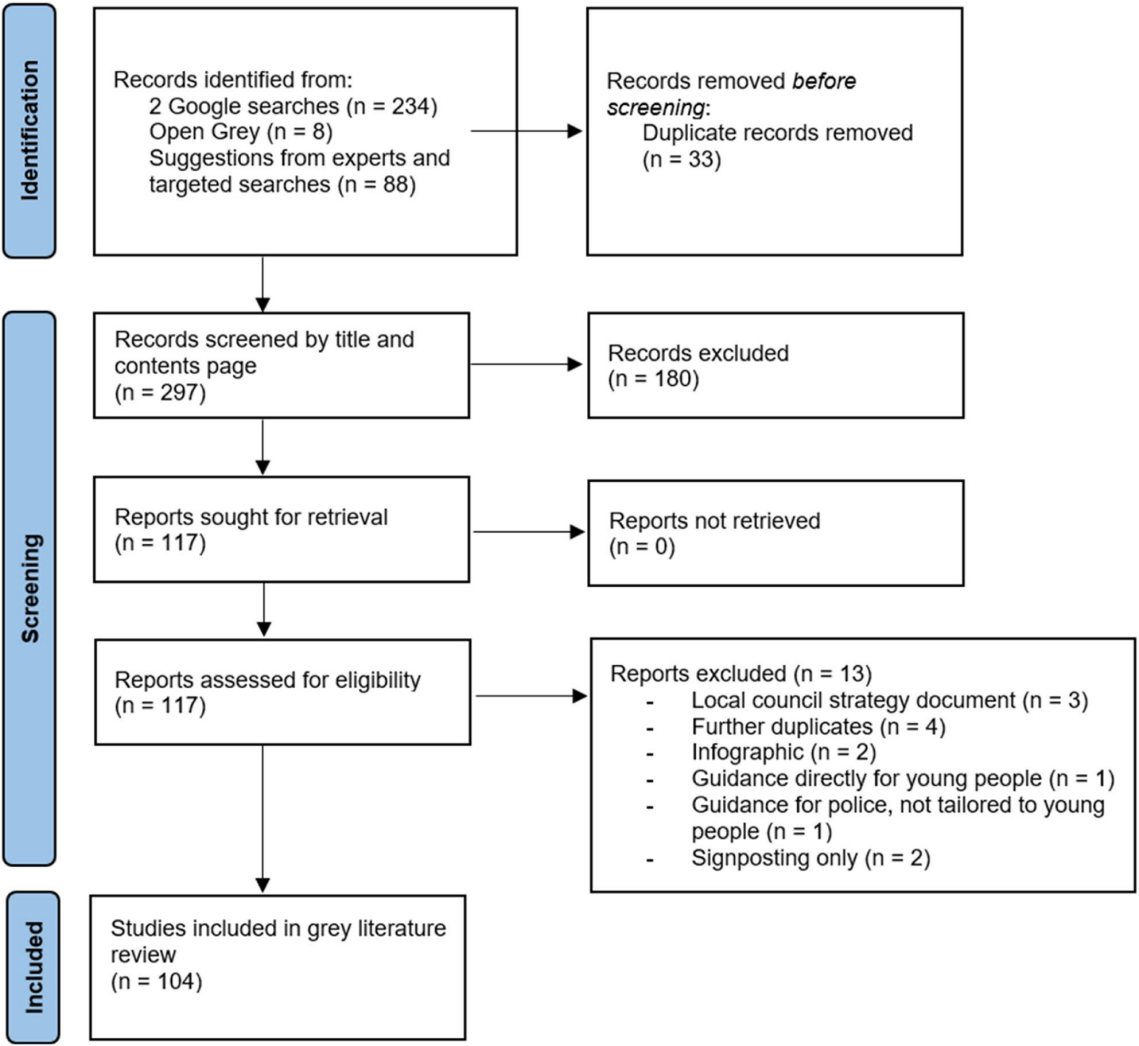
Prisma Flowchart

Figure 1 presents a flow diagram which is an adaptation of the traditional PRISMA flowchart and presents the number of results gathered from each of the search strategies. From the initial results obtained from Google (*n* = 234), Open Grey (*n* = 8), and experts and related targeted searches (*n* = 88), full-text review was conducted for 117 pieces of guidance. A total of 104 guidance resources were subsequently included in the review [32–134].

### Overview of guidance type and target audience

**Fig. 2:**
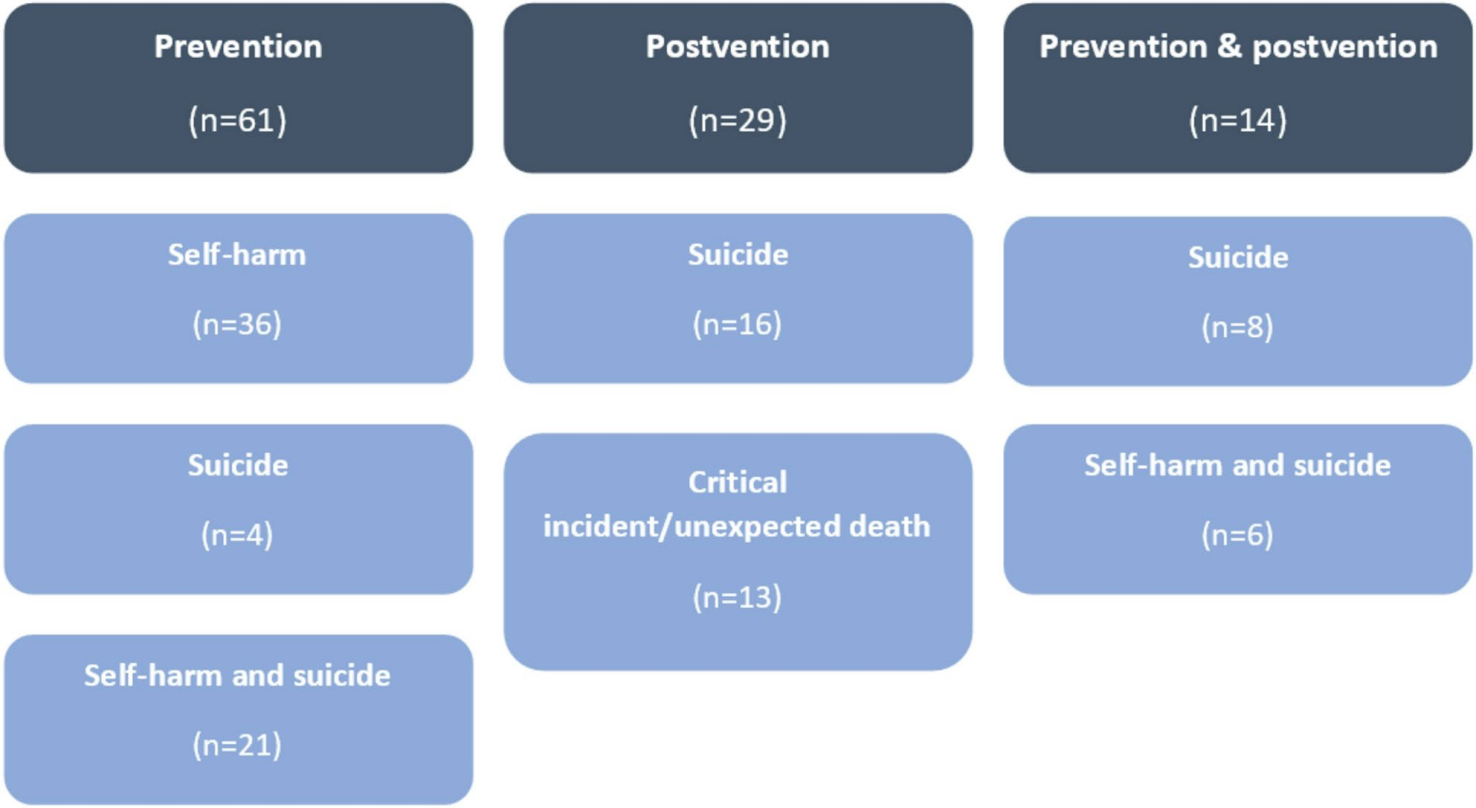
Overview of guidance type Critical incident =a single or sequence of incidents that are unexpected, cause severe disruption and are traumatic e.g. a fire or a hostage situation, unexpected death e.g. accidental or violent death

Figure 2 provides a visual summary of the guidance. Of the 104 resources, over half covered prevention only (*n* = 61), just under a third covered postvention only (*n* = 29), and the remaining 14 covered both prevention and postvention.

**Fig. 3:**
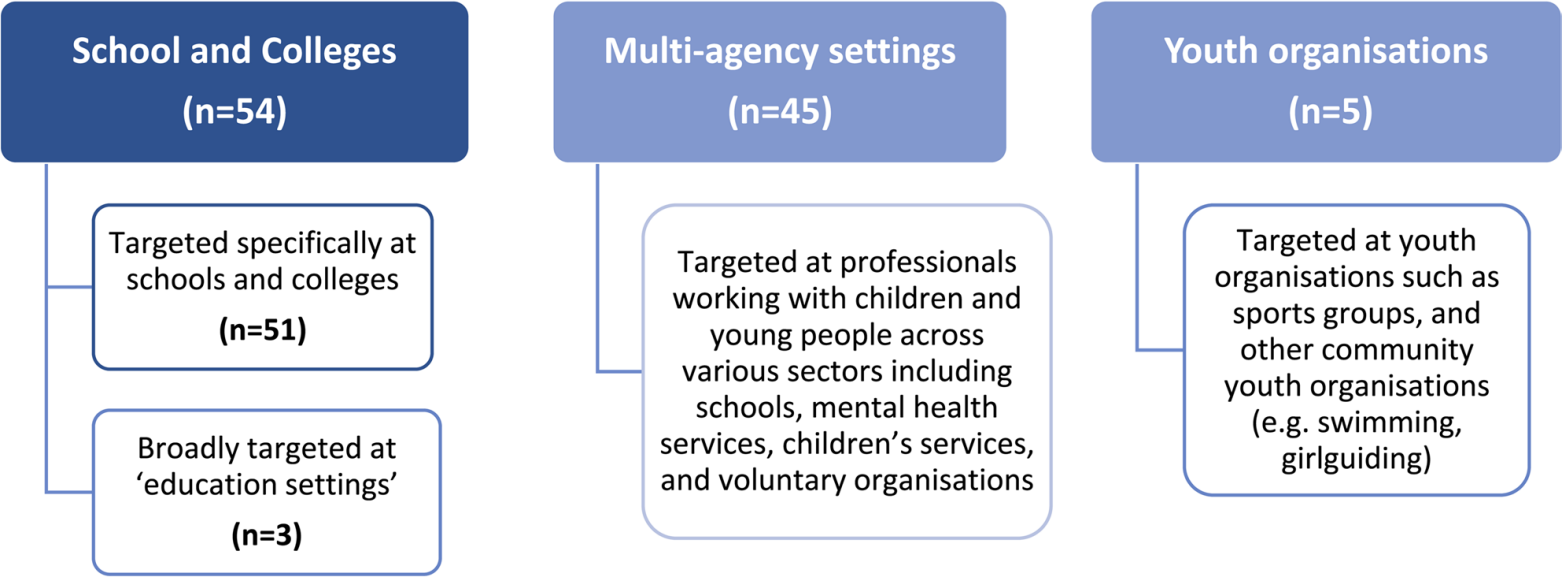
Overview of guidance by setting type

Figure 3 illustrates guidance by setting type. Just under half (*n* = 51) of all guidance resources were targeted specifically at schools and colleges with three being targeted more broadly at ‘education settings’. Forty-five guidance resources were targeted at multi-agency settings which were typically described as ‘professionals working with children and young people’ and included schools, colleges, youth organisations, mental health services, health visitors, police, children’s services and other voluntary and third sector organisations. Five resources were more specifically targeted at youth organisations (sports organisations in general [36, 134], swimming staff [128], Boys Brigade [125] and Girlguiding staff [48]). Of these youth-organisation specific guidance resources, none covered postvention and all five gave just broad and brief advice on self-harm in young people and were much shorter than multi-agency guidance and those targeted at schools/educational settings [128]. Given the number of multi-agency guidance documents found within this review, it is likely that youth organisations may use these resources as a source of support, however the multi-agency documents were quite broad and often covered a much wider remit than just self-harm and suicide (e.g. critical incidents more broadly) and therefore gave less detailed and contextualised advice and support.

### Guidance features

The content analysis revealed a total of 18 features. These were subsequently organised into three main sections of content: (1) Psychoeducation/Information, (2) Recommended Response and (3) Resources. Two additional guidance features were identified and categorised as ‘Additional Considerations’. A visual overview of the key sections of content and guidance features can be found in Table 1 which also includes the frequencies found in each type of guidance (self-harm only, suicide only and guidance covering both self-harm and suicide). The full guidance feature table can be found in *Supplementary Materials 3.* A narrative description of the features is provided below, as well as their prevalence in existing guidance.

**Table 1:** Overview of key areas and guidance features

Psychoeducation/information				Recommended Response				Resources				Additional Considerations			
	SH only^1^ (36)	S only^2^ (41)	SH&S^3^ (27)		SH only (36)	S only (41)	SH&S (27)		SH only (36)	S only (41)	SH&S (27)		SH only (36)	S only (41)	SH&S (27)
Definitions	100%	66%	96%	Conversations/language	58%	78%	78%	Templates/ checklists	58%	22%	41%	Tailored guidance (e.g. religion, LGBTQIA+, SEN)	6%	10%	33%
Prevalence & incidence rates	69%	34%	59%	Coping strategies/self-care advice	67%	39%	48%	Frameworks/diagrams	53%	27%	56%	Information on guidance development	17%	10%	7%
Risk factors, triggers & warning signs	92%	65%	100%	Whole school approach	22%	22%	33%	Information for parents/carers	50%	34%	52%				
Common myths & misperceptions	22%	17%	44%	Risk assessment	50%	51%	89%	Support/training for staff	44%	51%	41%				
Contagion/clustering	39%	41%	22%	Signposting	89%	80%	89%								
Internet & social media	22%	71%	52%	Legal issues	17%	15%	22%								
Confidentiality, consent & information sharing	69%	32%	40%	Postvention specific response (Funerals/memorials & handling the media)	0%	73%	0%								

^1^ SH only = documents that only included content on self-harm

^2^ S only = documents that only included content on suicide

^3^ SH&S = documents that included content on both self-harm and suicide

### Section 1: Psychoeducation and information

This section included the following six features;

#### Definitions

86% of guidance included definitions and general information on self-harm and suicide, for example: “*Self-harm is when somebody intentionally damages or injures themselves. It is often a way of dealing with difficult feelings*,* painful memories or overwhelming situations and experiences”* [36]. In addition, some guidance provided examples of self-harming behaviour (e.g. cutting, taking an overdose of tablets, burning) and information on the potential reasons for or functions of self-harming behaviour (e.g. distraction from problems, outlet for anger and rage, to not feel numb) [114]. Some guidance defined other common terms such as *“Suicidal ideation is a term used when people have thoughts or an unusual preoccupation with suicide. This can range from fleeting thoughts to detailed planning. Suicidal behaviour is a term used to describe non-fatal acts of self-injury that are motivated by suicidal intent.* [47]” Some suicide postvention guidance defined and described the process of grieving (e.g. a natural and necessary response to death).

#### Prevalence and incidence rates

53% of guidance included prevalence and/or incidence rates of self-harm and suicide which detailed how many young people are affected. Some guidance highlighted how prevalence of SH&S differs by gender, for example; “*At the time of review the suicide rate for males continues to be higher than that for females* [38].” Some guidance was created and intended for professionals in a specific area or region of the UK, yet few included local data around SH&S such as “*There were 11 suicides in Angus*,* 23 in Dundee City and 14 in Perth and Kinross in 2009*” [115]. Some guidance also included international data on SH&S [47].

#### Risk factors, triggers and warning signs

83% of included guidance contained information regarding risk factors, triggers and warning signs. For example, signs that a young person may be self-harming included spending more time alone and avoidance of activities that require changing clothes, whilst signs that a young person may be feeling suicidal included feelings of hopelessness and decreased interest in making plans for the future [36, 86]. Guidance from a suicide prevention charity (Papyrus) acknowledged that it is impossible to provide a definitive checklist of things to look out for to help identify an individual who is thinking about suicide because every young person is different, but encouraged professionals to explore any concerning behaviour or communication [39]. Risk factors identified which may make a young person vulnerable to self-harm included individual factors (e.g. low self-esteem), family factors (poor parental relationships and arguments) and social factors (persistent bullying), whilst triggers for self-harm included exam pressure, break up of relationship and significant trauma (e.g. bereavement) [114]. Some guidance also included examples of ‘protective factors’ which included the young person having good role models in the family, a stable and secure friendship group and good coping skills [100].

#### Common myths and misperceptions

26% of guidance included common misperceptions regarding self-harm and suicide, often presented as ‘myth busting’. This ranged from simply stating common myths to providing explanations as to why this was not true. Some myths that were often used as examples in guidance were that young people who self-harm are attention seeking, follow a goth sub-culture or are a risk to others [59]. Other guidance included sentences such as ‘*Talking about their self-harm or suicide thoughts or behaviours is NOT likely to (will not) put them at more risk. In fact*,* the opposite is true – talking and being listened to is a positive and saving process* [51].’

#### Contagion/clustering

36% of guidance discussed issues with contagion and clustering. Self-harm guidance sometimes raised awareness that students in the same peer group may be harming themselves. One guidance was specifically focused on responding to a potential cluster of suicides and noted that following the identification of a suicide cluster there is the need for significant specialist input and a dynamic multi-agency response to assess further young people at risk of suicide [81].

#### Internet/social media

49% of guidance included information regarding the likely impacts of the internet or social media on youth self-harm and suicide. Social media pressure came up as a risk factor for self-harm whilst suicide-related internet use was highlighted as a warning sign of suicidal ideation and suicide, as well as being linked to why people take their own life [60]. Guidance often referred to the dual role of the online world on the mental health of young people, highlighting both the potential for harm and the potential benefits of use. Benefits referred to in guidance included access to and delivery of therapy, and reduced social isolation, whilst risks relating to suicidal behaviour included online bullying and normalisation [86].

Some self-harm guidance raised awareness that young people may access images of self-harm on social media, share details or images of their own self-harm [133], or perhaps would use the internet or social media to look up ways to self-harm [96]. Guidance also referred to the internet/social media posing a risk by promoting different methods and normalising self-harm [91] whilst “*social media portrayal may glamorise self-harm and elicit “copy-cat” responses”* [100].

Suicide postvention guidance highlighted that following a suicide, young people turn to social media for a variety of reasons including “*sending news out about a death (both accurate and rumoured)*,* posting online messages (both appropriate and inappropriate) and calling for impromptu gatherings or creating virtual memorials*” [37]. Postvention guidance also often detailed difficulties surrounding news of a suicide circulating on social media before schools/organisations had informed their own pupils or staff. Other postvention guidance stated potential advantages (brings people together, keeps memories alive, fundraising campaigns) and disadvantages (negative comments, false information, no immediate support) of social media use during bereavement [61].

Although almost half of guidance resources included information on the impact of the internet/social media, few of these provided guidance on what education/youth settings should do in response to the issues raised. The guidance resources that did provide more detail on this suggested some points for consideration including to set up a memorial page that is monitored by students or support staff, educate the community on safe messaging and to encourage students to send positive supportive messages and report any concerns to staff [39].

### Section 2: Recommended response

The second section within the guidance focused on how to respond to disclosures of self-harm or suicidal thoughts and how to respond to a death by suicide. This section included the following features;

#### Conversations/language

A majority (71%) of guidance included how to talk to young people about self-harm and suicide. This frequently included ‘top tips’ or ’Do’s and don’ts’ for talking to pupils, for example; take a non-judgemental attitude towards the young person, be an active listener and resist the temptation to tell them not to self-harm again [88]. Some guidance provided conversation prompts and questions for professionals to use when initiating conversations e.g. “*I wonder if anything specific has happened to make you feel like this or whether there are several things that are going on at the moment.?*” [95]. Guidance also sometimes discussed definitions or justifications for particular language used within the document, recognising that some language can be stigmatising and that varying definitions can be used creating a lack of consistency. For example, guidance highlighted that using the phrase ‘committed suicide’ can be unhelpful and can perpetuate stigma due to the implication that suicide is a criminal activity (which it has not been in the UK since 1961) and suggested language such as ‘ended their life’ or ‘killed themselves’ should be encouraged [39].

#### Coping strategies/self-care advice

In total, 50% of guidance included information on coping strategies/self-care guidance for young people, who were self-harming and/or experiencing suicidal thoughts. This often took the form of lists of possible alternative coping strategies or distraction techniques including writing negative feelings on a piece of paper then ripping it up, going for a walk in nature and talking to a friend or family member [104]. In other guidance, it was simply acknowledged that the professional should explore other coping strategies with the individual who is self-harming or has suicidal thoughts. Suicide postvention-only guidance focused on how to support the bereavement needs of young people after a death by suicide. For example, The Pan-Sussex Toolkit included an appendix of ‘*Suggested Activities for a Bereavement Support Room*’ which suggested provision of a safe, supervised room where students’ grief and needs can be expressed, responded to, and monitored [82]. The activities included breathing techniques, creating memory jars and boxes, and mindful colouring as well as asking the students what they would like to do and what would help them.

#### Whole school promotion of good mental health and well-being

25% of guidance referred to general promotion of good mental health and well-being as a whole school or organisation approach, which was often not specific to self-harm or suicide but focused on general prevention of mental health difficulties more broadly. Guidance sometimes referred to resources for teachers to use relating to students general mental health and well-being, for example information on ‘Mentally Healthy Schools’ initiatives or advice for Personal, Social, Health and Economic (PSHE) lessons (PSHE is a school curriculum subject in England that helps children and young people develop the skills and knowledge they need to be safe, healthy, and prepared for life). Some guidance had a section specifically relating to ‘a whole school approach’, for example highlighting that ‘the promotion of pupils’ positive mental health to build resilience is a core responsibility’ [77]. One piece of guidance focused specifically on a whole school approach to self-harm awareness and training, this guidance noted that not all school staff need to be experts but it is important to encourage a baseline understanding of self-harm in all staff [34].

#### Risk assessment

61% of guidance included information on assessing risk and risk assessment templates. Within self-harm and suicide prevention and intervention guidance, this was often related to assessing immediate risk to the young person following a disclosure or identification of self-harming behaviour or suicidal ideation. Guidance included factors that increased the risk (e.g. if there has been an increase in self-harm behaviour) and how to mitigate immediate risks by ensuring any physical wounds are treated. Prevention guidance often discussed a spectrum of risk, categorising this as low, medium, high and emergency [78] or used the THRIVE model to categorise risk into ‘coping/getting help’, ‘getting more help’ and ‘getting risk support’ with the suggested pathways of support depending on the category [69]. Where guidance did not have specific risk assessment templates, it was often noted that it is important for the organisation to have a risk assessment in place.

Within postvention guidance, risk assessments focused on the identification and assessment of risk in young people who are more vulnerable or in need of more support following a death by suicide, also in relation to preventing contagion or clustering (further suicides).

Relating to postvention risk assessment and an immediate response, there were relatively few guidance resources that provided a timeline of response. A few examples include ‘crisis response steps’ within Portsmouth City Council’s Suicide Prevention and Postvention Protocol for Schools and Colleges [111]. Another example that clearly listed a time sensitive response plan was a Pan-Sussex toolkit co-developed by schools, colleges, the Community & Voluntary Sector, and the Police which included guidance for ‘immediate response’, ‘the first day of school’, ‘the first week’, ‘the first month’ etc [82].

#### Signposting (organisations, charities, healthcare providers and individual contacts)

85% of guidance included signposting information. Typically, this was in the form of signposting to national charity websites and helplines but sometimes guidance signposted to specific interventions, for example Samaritan’s ‘Step by Step’ suicide postvention support.

As well as generic signposting to charity/organisation websites, some guidance also included named individual contacts (e.g. a local educational psychologist), as well as signposting to local agencies and organisations which often included detail on how to make a referral.

In total, over 350 national and local organisations were referred to. Table 2 shows the top 25 organisations most frequently referred to and the number of citations to each.

**Table 2:** 25 organisations most frequently signposted to within the included guidance

Name of organisation	Number of guidance resources citing organisation
Samaritans	66
Young Minds	66
Childline	59
Papyrus	45
National Self-Harm Network	32
Mind	30
CAMHS	26
Cruse Bereavement Care	24
Alumina (previously Self-Harm UK)	23
Winston’s Wish	19
Hopeline UK	14
NSPCC Counselling Support for Children	14
Child Bereavement UK	14
LifeSigns	14
The Mix	14
Kooth	13
CALM (Campaign Against Living Miserably)	12
Harmless	12
Survivors of Bereavement / Suicide	12
Breathing Space Helpline	11
NHS111/NHS24	11
Self Injury Support	11
Calm Harm (app)	10
Royal College of Psychiatrists	10
The Compassionate Friends	9

#### Confidentiality, consent and information sharing

56% of guidance included information regarding confidentiality procedures, gaining consent and sharing of information. Points frequently raised included not making promises of confidentiality to young people [69] and the need for parental consent when arranging treatment/referrals for young people under the age of 16. Guidance often advised to involve the young person wherever possible, and that staff should tell young people when they must share information without their consent [38].

#### Legal issues

Information surrounding legal issues appeared in 15% of all guidance. Detailed information relating to legal issues was predominantly in relation to postvention guidance, which explained things such as the role of the coroner and the inquest process, for example highlighting that those known to the deceased may be called to give evidence [49]. Legal issues in prevention guidance mostly covered legislation that is relevant to confidentiality, consent and information sharing (e.g. ‘The Mental Capacity Act 2005’, ‘The Age of Legal Capacity (Scotland) Act 1991’, ‘The Data Protection Act 2018’) as well as information on when and how to follow child protection procedures.

#### Postvention response

Of the postvention specific guidance, 58% contained information on handling the media. Suggestions included assigning a member of staff to be a nominated media liaison representative [45], using an approved and prepared statement [33] and advising students to avoid interviews with the media. Other guidance referred users to their local authority for support with communications [111] or to Samaritans’ ‘Handling the Media’ document [135]. One piece of postvention guidance also discussed the possibility of building relationships with some reporters with a view to negotiating with them about how the incident can most sensitively be reported [132].

12 out of the 43 (28%) guidance resources that contained postvention information included advice about arranging funerals, memorials or remembrance events after a death by suicide. This commonly included acknowledging the importance of such events for the grieving process among the community, whilst needing to avoid romanticising, glamorising or sensationalising death by suicide. Guidance often highlighted the need to have a carefully considered designated place for flowers etc. to be left and for there to be an end date for these displays (two weeks after the death was often suggested). Consulting with the parents and/or wider family of the deceased was often highlighted as important to establish their wishes and views on the funeral and potential memorials. Content regarding funerals often described the varying cultural and religious beliefs and norms of funerals (see 4.1 below).

### Section 3: Resources

The third section covered resources organisations could use to support young people. These tended to include more practical steps for staff to follow or carry out. This section included four features detailed below.

#### Templates/checklists

39% of guidance provided templates and resources for schools/youth organisations, typically found in the appendix These included checklists (e.g. protective/risk factor checklists or risk assessment/action checklists), sample safety plans, letters to the media, incident report forms and ‘safety net’ templates for young people to complete to encourage them to think about their support networks. Some also included detailed case studies. In their self-harm guidance for staff in schools and residential settings, for example, the Oxfordshire Adolescent Self-harm Forum guidance [91] provided an example letter that a school can send to a parent following a meeting about their child’s self-harm (see *Supplementary Materials 4)*. The Ollie Foundation guidance for educational settings [49] following a suicide or sudden death included many of the aforementioned resources and templates, including an example ‘vulnerable students log’ to be used by schools to highlight and make other staff aware of those who may need additional support and/or be at higher risk of imitation behaviour or suicide (See *Supplementary Materials 5*).

#### Frameworks/Diagrams

43% of guidance included frameworks and diagrams to support schools/youth organisations. Common examples included fact sheets, ‘Dos and Don’ts’, diagrams such as care pathways and referral pathways, and flowcharts of response or intervention pathways. Example diagrams are shown in Supplementary Materials 6.

#### Information for parents/carers

44% of guidance included information for organisations to share with parents/carers. This included specific signposting tailored for parents and carers, as well as information, sample letters or fact sheets and advice on how to start conversations with their child about self-harm or particular language to avoid.

#### Support/training for staff

46% of guidance included information on how to support staff, this included both generic support information, specific resources for staff, or training for staff such as the Applied Suicide Intervention Skills Training (ASIST, https://www.asisttraininguk.co.uk/).

### Section 4: Additional considerations

We also grouped two features into an additional considerations category which had a lower prevalence across all of the guidance resources reviewed which included tailored guidance and information on guidance development.

#### Tailored guidance for specific populations/vulnerable groups (culture, religious, LGBTQIA+, SEND)

14% of guidance included tailored information for specific populations; most commonly this consisted of young people with special educational needs and young carers. Often this did not constitute tailored intervention or response, rather raising awareness that response would need to be tailored. Some suicide postvention guidance referred to the importance of cultural and religious beliefs when responding to a death by suicide and included information on specific religions and cultures (e.g. gypsy and traveller communities) and their likely practices and views regarding death and funerals.

#### Information on guidance development

12% of guidance detailed how it had been developed. Multi-agency guidance typically detailed that it had been developed from published reports, policy guidance and guidance developed for the NHS or wider council remits. Other documents detailed that they had used example guidelines (e.g. from a charity) to develop their own tailored local version [132]. Very few guidance resources detailed that they had been designed in consultation with young people. One guidance resource specifically dated young person involvement [132] and another appeared to include resources more tailored to young people, for example through used of imagery (See Supplementary Materials 7). This particular piece of guidance was developed by the Oxfordshire Adolescent Self-harm Forum which consists of a steering group of a range of experts within the field including psychiatrists, educational psychologists, clinical psychologists and school health nurses [91].

### Gap analysis findings

**Fig. 4:**
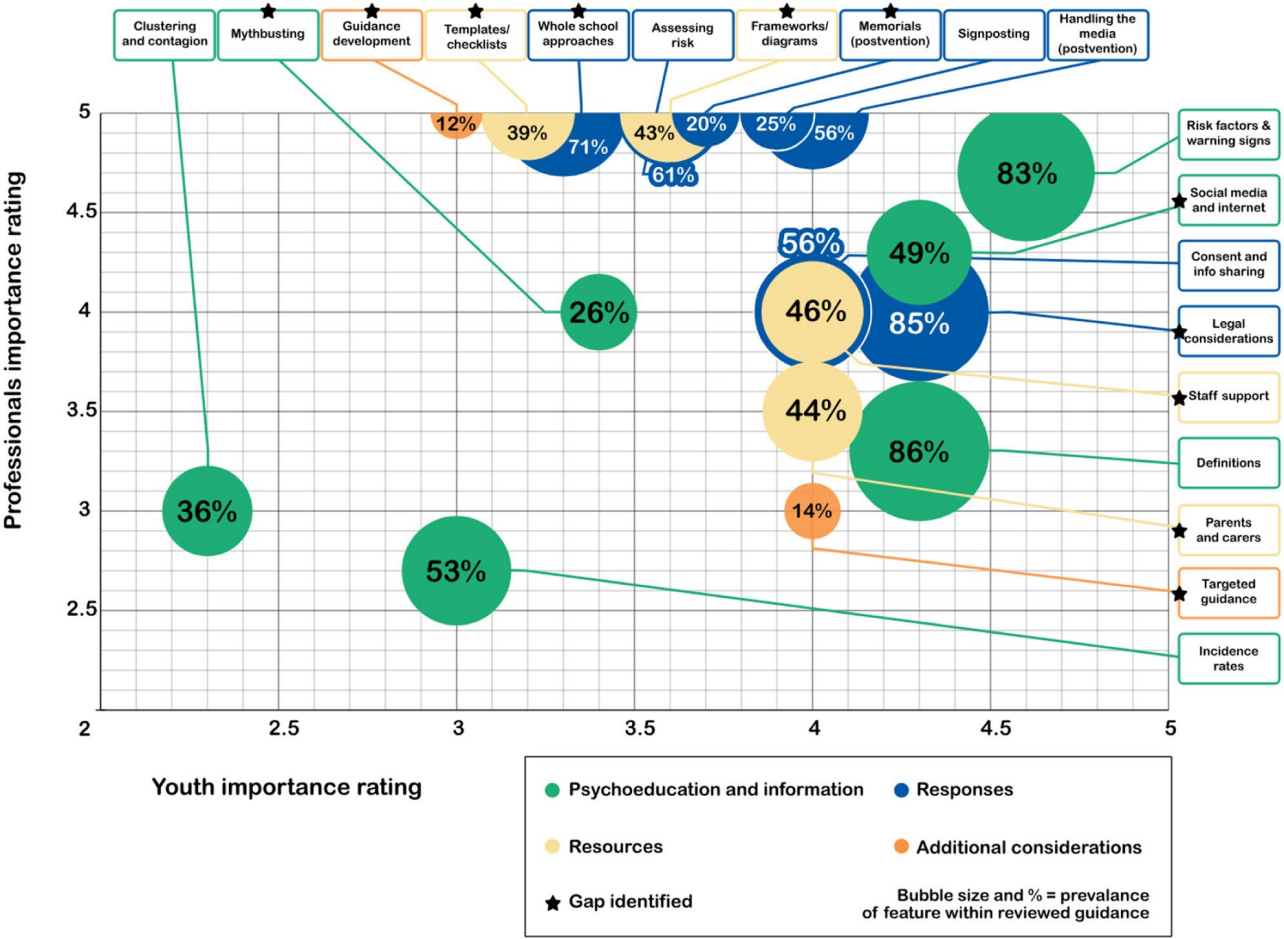
Gap Analysis Bubble Plot

Figure 4 is a bubble plot of our gap analysis which illustrates each feature with its prevalence found in existing guidance, professional and youth ratings. We synthesised these multiple sources of evidence, including qualitative statements from our advisory groups, to identify features that appeared to be important but underrepresented in current guidance. Features marked with a star were identified as a gap within existing guidance. Key findings from our gap analysis are also described in further detail below.

### Psychoeducation/information

The top-rated feature within this section by both young people and adult stakeholders was risk factors, triggers and warning signs, which was not identified as a gap as it appeared in 83% of guidance documents. The gaps identified within the psychoeducation/information section were ‘common myths and misperceptions, and ‘internet and social media’ which were both rated as important by stakeholders but found in less than half of guidance. Relating to common myths and misperceptions, the young person stakeholder group suggested that it is important for professionals to be aware that it is not always the ‘stereotypical’ person that experiences self-harm or suicide. Relating to internet and social media one professional stakeholder noted that they felt young people were already aware of the positives and negatives around social media whereas professionals are often playing catch-up, therefore information relating to the internet and social media was particularly important within guidance for professionals.

The lowest average rated feature was contagion/clustering. There was a general consensus among stakeholders that these terms were unhelpful or too simplistic. Adult stakeholders also highlighted the importance of nuance with these concepts. Another adult stakeholder raised concerns regarding guidance containing information on contagion; “*who would the conversation about ‘contagion’ be held with? We need to be careful not to encourage others to consider taking their lives but steer them away from/prevent contagion*”. An additional reflection from adult stakeholders was the concern regarding contagion and clustering and whether this had implications regarding peers supporting one another. Although some guidance resources did refer to peers, this tended to be framed as a negative for example listed as a trigger for self-harm (to identify with a peer group), as well as references to peers relating to contagion and social media. Typically, guidance referred to how adults, parents and caregivers could speak to young people about self-harm or suicide, guidance resources seemed to lack focus on how young people could support each other.

In terms of key comments from stakeholders regarding improvement of the psychoeducation/ information section, an adult stakeholder highlighted that although definitions are important, they are constantly changing and therefore there needs to be adaptation and flexibility with terminology around self-harm and suicide. This also reflected the importance of guidance needing to be frequently reviewed and updated. Adult stakeholders also discussed how national prevalence statistics do not always reflect regional disparities and therefore would be less helpful in understanding local data and local priorities. A young person stakeholder also suggested that prevalence/incidence rates could be more helpful if categorised by different groups for example prevalence by age, gender, location and specific groups such as neurodivergent young people.

### Recommended response

The gaps identified within the recommended response section were general well-being promotion in schools, legal issues and guidance on funerals and memorials. A young person stakeholder noted that although well-being promotion in school is important, ‘wellbeing’ was something they felt was a longer-term outcome and therefore for a young person struggling with self-harm or suicidal thoughts, it may not appear immediately helpful or relevant. Adult stakeholders noted the importance of guidance around legal issues for staff, particularly around suicide postvention guidance but also noted the need for support for those involved. Another stakeholder noted the implications around the requirement not to talk about a suicide due to waiting for a coroner’s inquest and the impact this has on providing support to those involved. Relating to funerals and memorials, one professional highlighted the importance of acknowledging what a family/community culture may be around funeral practices whilst recognising more diverse ways of approaching memorials.

Coping strategies/self-care advice had the largest difference in ratings between adult stakeholders and young people with young people rating it as very important (4.7/5) but adult stakeholders only rating it 2.5/5. One adult said that there is *“lots of great generic [self-care] information already out there*,* would be great to highlight those but delve deeper into the things less spoken about”*. Other stakeholders noted that these tend to be very general and not specific to self-harm for example ‘have a bath’ or ‘have a cup of tea’ which may feel unhelpful or unfocused for a young person self-harming.

In terms of recommendations to improve future guidance, one professional highlighted the importance of staff feeling confident when talking with young people and that following a framework or piece of guidance was insufficient.

With regard to postvention guidance, one adult stakeholder noted the importance of “awareness of individuals who are more vulnerable following a death by suicide” and noted “The Circles of Vulnerability model” which is a resource that can help identify the level of emotional impact a traumatic event has on a community and can be used to assess the impact of a suicide death on a school community. This has been used relating to suicide contagion within Australian guidance by Headspace.

In relation to signposting, one adult stakeholder highlighted the importance of understanding local support and suggested resources such as Hub of Hope which allows you to search for mental health support by postcode as well as filtering by support for young people only. This resource was not identified in any of the guidance included within the review.

### Resources

An important finding from the gap analysis is that all four key features within the resources section were identified as a gap which reflects the lack of more practical resources found in guidance noted within the results section.

Although adult stakeholders rated templates/checklists and frameworks/diagrams as the highest importance (5/5), young people rated these two features much lower. One stakeholder also noted that “a framework is only as good as the people using them”.

A lack of guidance was identified for parents/carers for both self-harm and suicide guidance. Particularly for suicide postvention guidance, there was a lack of guidance relating to supporting wider family members, including information on how to best support siblings after a death by suicide and the implications of this if they are within a different school for example. Professionals highlighted that support for parents/carers could be a set of guidance in itself, highlighting the importance of this gap. Young people suggested specific signposting for parents (which some guidance did include) and inclusion of the term ‘guardian’ in addition to ‘parents and carers’.

The importance of supporting staff well-being and providing training for staff was rated as 4/5 by both professionals and young people. One young person recognised the importance for staff to have access to mental health professionals to support them and also highlighted the need for training so staff can be better prepared for the impact on their own well-being. Adult stakeholders highlighted the importance of regularly revisiting this training, for example on an annual basis.

In conversation with our YPAG, one member suggested that the use of case studies are missing from current UK guidance. Case studies appeared in very few guidance resources, a few examples included written scenarios of an example conversation with a young person about self-harm and a case study of somebody who had attempted suicide written by their mother. The young person in our YPAG raised this as something they would find important for guidance to allow both young people and professionals to reflect on different experiences and real-life examples. This could also be linked to peer support if real life examples include similarly aged young people.

### Additional considerations

Both ‘additional considerations’ were identified as a gap within current guidance. While tailored guidance for specific populations/vulnerable groups was identified as a gap, it was recognised that it would be difficult to write an exhaustive list of considerations for particular groups/populations. One young person suggested that future guidance could provide digital links to different groups and specific considerations so professionals can access additional information without the guidance becoming too long. One stakeholder also asserted that if a whole school approach is adopted and the school knows their community, considerations for particular groups or vulnerable students would be implicit.

Information on guidance development was the least prevalent feature found within current guidance but among the highest rated by adult stakeholders. Although ranked slightly lower by young person stakeholders (3/5), one young person argued that information about where the information was sourced is almost as important as the guidance itself.

Table 3 suggests a number of specific ways future guidance could be strengthened based on the key findings and gap analysis presented within this review including qualitative comments from our stakeholder groups. These are subsequently discussed in further detail in relation to the current literature surrounding prevention and postvention guidance for education and youth settings.

**Table 3:** Key considerations for future guidance development

Key recommendations to improve future guidance
Guidance for non-educational settings (e.g. youth organisations)
More procedural guidance for response after a suicide
Clearer link between guidance and evidence-base
Guidance co-produced with young people and staff working with young people
Targeted signposting/resources specific to local contexts
More information for how organisations can support parents and families with conversations around self-harm or following a suicide
More on how to inform the wider community and wider community support
How peers can support each other
A focus on ensuring cultural sensitivity
Tailored guidance for specific communities or vulnerable groups (religious groups, different cultures, LGBTQIA+, SEND)
More guidance around digital safety and social media to include both benefits and harms

## Discussion

This systematic review of publicly available grey literature found 104 self-harm and suicide prevention/postvention guideline resources targeted at educational or youth organisations. Prevention-only guidance was the most common (59%), compared to just 28% providing postvention-only guidance and only a further 13% providing both prevention and postvention guidance. Guidance typically consisted of three areas: (1) Psychoeducation/information; (2) Recommended Response; and (3) Resources. We also identified two additional areas much less commonly included, which were tailored guidance for specific populations and/or vulnerable groups as well as information on guidance development.

Prevention guidance most commonly included information on general well-being promotion in school/settings, how to approach talking about self-harm and fairly broad signposting to relevant websites and charities (often national). Overall, prevention guidance tended to focus on information giving and included less about ‘how to’, for example how to act or what steps to take in response to self-harm disclosures, or suicidal ideation. Although postvention guidance did include more practical and step-by-step responses, only 21% of these documents contained templates and resources for staff to use and only 26% contained frameworks and diagrams. Signposting to other organisations was a core feature of nearly all guidance, with sometimes 20–30 organisations being signposted to However, there was a lack of detail on specific assistance these organisations could provide, which places onus on education and youth settings to research in order to determine their appropriateness.

A key gap identified in this review was the lack of clarity in many guidance documents regarding the evidence underpinning specific recommendations. Several documents provided practical advice without clearly citing empirical studies, systematic reviews, or established theoretical frameworks, making it difficult for users to assess the strength, relevance or currency of the information provided. Transparent sourcing of evidence is a core principle of evidence-informed practice and is particularly important in the context of self-harm and suicide, where inaccurate or outdated information may carry potential risks.

Recent research has highlighted challenges in translating suicide prevention evidence into policy and practice, including variability in how evidence is interpreted, synthesised and presented in guidance materials [136, 137]. There have also been recent calls to move towards focusing on safety rather than risk assessment in suicide prevention [138], a key point demonstrating the need for guidance to be aligned with current evidence given that many guidance documents included within this review included a focus on risk assessment and provided risk assessment templates. Clearer links between guidance and its evidence base, such as explicit referencing, differentiation between empirical evidence and expert consensus, and acknowledgement of uncertainty, may support greater confidence among staff and facilitate more consistent implementation. Strengthening these links should therefore be a priority for future guidance development and revision.

Another key finding of interest was the lack of guidance specifically tailored to youth organisations. Although there were many broader multi-agency guidance documents for a range of professionals working in settings with children and young people, there were only five pieces of guidance focused on specific youth organisations (covering ‘sports organisations’, swimming, Girlguiding and The Boys Brigade). It is important for future guidance to be inclusive of staff working in non-education settings, particularly given the rising number of young people who are not in school [139] as well as young people confiding in or help-seeking from adults outside of school settings given known issues relating to young people trusting school staff when disclosing self-harm [140]. There is a need for further research into what happens when self-harm, suicidal ideation or a suicide occurs in non-school settings and what adults working in these settings would like to see and find helpful in tailored guidance resources.

Some of the most relevant published literature to discuss in relation to our review are two recent Delphi studies that are gaining consensus on important features of guidance resources. There were a number of similarities between our postvention guidance findings and key findings identified within an Australian Delphi study developing postvention guidance for secondary schools [19]. Guidance for staff around the implications of the internet and social media in SH&S support was identified within our review which was also discussed in the Delphi study, mainly in relation to guidance on memorial pages set-up on social media. Interestingly, the experts within the study did not endorse this practice and therefore online memorial pages were not recommended within the guidance produced. Avoidance of students romanticising or glamorising a death was also highlighted in the Australian Delphi study. Providing support to staff was an identified gap within this review which was highlighted within the Australian Delphi study regarding giving staff opportunities to ask questions and express their own reactions and grief as well as providing information on relevant support networks. We know that there is a need for good quality self-harm training for UK school staff with a recent study demonstrating that only half of teachers had received training on adolescent self-harm and only 22% rated the adequacy of this training as high [141]. The difference in adult and young person ratings revealed in our gap analysis relating to staff using templates and checklists could also reflect the lack of confidence professionals feel in supporting young people and the need for staff to follow a process/checklist for reassurance compared to young people perhaps seeking more informal and relaxed conversations.

The Australian Delphi postvention study also highlighted the importance of identifying and supporting ‘at risk’ students after a death by suicide which included monitoring suicide-related rumours, notes or messages amongst other students as well as monitoring student absences [19]. This related to a point raised by our adult stakeholders regarding the importance of knowing which students are vulnerable or more at risk following the death of a student. Although guidelines often included information on risk assessments, specific guidance on how to monitor the most vulnerable students after a death by suicide was missing from UK guidance.

Another key area of importance raised from this Delphi study was the need to inform the wider community of a death by suicide, which included liaising with local mental health services, community teams and local sports groups. This wider community emphasis was not a key focus within the UK guidance identified in this review and seems an important aspect to be considered in future postvention guidance. This also links back to the general lack of detailed information given to organisation to enable them to support parents and families in both prevention and postvention guidance.

In terms of how our findings related to a recent New Zealand Delphi study developing guidelines for self-harm prevention in schools [21], Meinhardt et al. revealed the importance of incorporating the student voice and strong connections between students and families (aspects both missing from current UK guidance) as well as providing role specific guidance for school staff at different levels which current UK guidance did not typically detail. Interestingly the sections of the guidance mapped out from the New Zealand study differed to the typical structure of current UK guidance, their five sections included working together to support students, responding to a disclosure/incident of self-harm, what to do after a disclosure/incident, how to provide ongoing support and how to support staff. In comparison to the key sections found in UK guidance, the sections outlined from the Delphi study provide more of a focus on connected and collaborative provision and immediate and longer-term support which is an important consideration for how future UK guidance could be organised.

A large international Delphi study exploring the design of school-based awareness programs for suicide prevention [142] noted the importance of peer support, particularly given peers often only disclose suicidality to their peers [143]. Peer support is an important aspect to be considered for future guidance resources and it will be important to hear whether young people and professionals view peer support as an important topic to be included in future guidance, particularly given the influence of peers within this age group as well as existing evidence on peer-based approaches around self-harm and suicide [144–147]. However, given concerns around contagion amongst peers both detailed within current UK guidance and addressed by our adult stakeholders, there is also a need to ensure safeguarding around peer support, as well as avoiding young people feeling they are responsible for peer disclosures. A recent UK study demonstrated the acceptability of peer support for self-harm with benefits including a sense of community, empowerment, and access to information and support [145]. One associated risk discussed in this study was peer support triggering self-harm and mitigations for potential risks included using professional facilitators for groups, trigger warnings, and regular supervision and training for peers.

The comparison with Australian Delphi studies, undertaken in a broadly comparable national context, highlights the need for UK postvention guidance to move beyond high-level principles towards greater practical and procedural clarity. In particular, UK guidance would benefit from more detailed direction on how schools should identify, monitor, and support students at heightened risk following a suicide, rather than relying primarily on generic risk assessment processes. There is also a clear need for enhanced training and structured support for school staff, both to build confidence and to address staff well-being. Furthermore, UK policy could place greater emphasis on coordinated, whole-system responses, including explicit guidance on working with families, local mental health services, and community organisations after a death. Finally, while peer support shows promise within the UK context, its inclusion in future guidance should be accompanied by specific safeguarding measures, supervision, and role clarity, ensuring young people are supported without being placed under undue responsibility. These findings point to a clear need for an in-depth UK Delphi study to build expert consensus on specific, practical self-harm and suicide prevention and postvention actions that are tailored to the UK educational and policy context.

A bigger focus on how organisations can better support parents and families is an important consideration for future guidance, given that we know parents and carers can feel anxious and sometimes guilty when they find out their child is self-harming [148]. More targeted support for families also extends to siblings, with parents reporting how siblings can be upset and stressed as well as concerned about stigma at school [148]. One qualitative US study that spoke with parents with children at risk of suicide revealed the importance of ongoing support for parents and families and also suggested a need for specific training for school staff on parent engagement [149].

In addition to involving parents and families, another aspect that was missing in existing UK postvention guidance, but raised within the Australian Delphi study, was the importance of informing the wider community and organising wider community support [19]. In this review, guidance resources typically only covered communicating with those immediately impacted such as students, school staff and families. However, expert recommendations from the Australian Delphi study highlight the importance of organisations seeking external support and alerting local postvention support as soon as possible to ensure a joined-up approach e.g. local mental health services and community response teams. Additionally, this guidance suggested informing principals of neighbouring schools as well as the siblings’ schools.

Another key gap found in this review was the lack of tailored guidance to specific groups, for example ethnic minorities, religious groups, LGBTQIA+ communities. Current guidance therefore may not be sensitive to particular groups and communities which could be problematic given that we know some of these groups are more vulnerable to or at risk of self-harm or suicide, for example LGBTQIA+ communities and neurodiverse young people [150, 151]. More research is therefore required with vulnerable groups to better understand preferences around language, help-seeking and whether key features of guidance may differ for these different groups. Although it may not be feasible to have a large number of individual guidance documents tailored to different groups, future guidance could include a section on vulnerable groups to include sensitivities around things such as language, cultural norms and religious beliefs. Some guidance did encourage staff to acknowledge the individual child and their particular circumstances. All guidance should also provide information on how to handle self-harm/suicide disclosures while being sensitive to the cultural, religious, and individual beliefs of young people and their families. Different cultures have different views on mental health and suicide [152], and these should be respected and considered when implementing prevention and postvention strategies.

With the increasing use of digital platforms amongst young people and rising concerns about social media facilitating self-harm and suicide [153], it would be beneficial to include more concrete guidelines on online safety and the role of social media in self-harm and suicide, both positive and negative including both how young people could use social media for support but also how unhelpful content could appear if a young person may be searching for self-harm or suicide related content.

Another important consideration for future guidance is information on how to collect pertinent data and evaluate the effectiveness of the organisation’s response and the support provided. This could help settings continuously improve their strategies and be better prepared for potential future incidents. This would also encourage education and youth settings to regularly update their guidance.

### Limitations

Although we approached this grey literature review in a systematic way using known methods [26, 27], we acknowledge that the internet searches will not capture all publicly available guidance. It is also likely that a number of educational settings and smaller local youth organisations may have their own guidance for staff that is not publicly available, which was therefore not included within this review. Further, it is important to reiterate that this review did not include individual school protocols or policies. Additionally, due to this being a grey literature review, we were not able to formally assess the quality of guidance contents. Further research is necessary around the evidence-base regarding effective prevention and postvention approaches.

This review focused on mapping the scope, content and thematic coverage of UK grey literature guidance, rather than evaluating the scientific accuracy or evidential alignment of individual statements within documents (e.g. prevalence estimates or risk factors). Assessing accuracy would have required a different methodological framework with predefined criteria and was therefore beyond the scope of this review. Future research could examine the consistency, accuracy and evidential basis of key messages within guidance to support confidence in its reliability and use in practice. Additionally, the absence of a formal quality assessment means that the review does not distinguish between guidance documents based on their methodological rigour or evidential underpinning, which limits the generalisability of findings and requires caution when interpreting the relative strength or reliability of specific recommendations.

The advisory group input was based on small group sizes and was included as an additional, exploratory component to complement the content analysis rather than as a formal consensus-generating method. While there was evidence of thematic convergence across many guidance components, some variability in ratings was observed, particularly between young people and adult groups, reflecting differing perspectives and priorities. The guidance contained within this review is largely aimed at professionals working with young people rather than young people themselves which is likely to explain some of the discrepancy between ratings. Although the young people’s advisory group included participants with diverse cultural backgrounds, geographical localities, and lived experiences of self-harm and suicidality, findings from this component should be interpreted cautiously and not assumed to be representative of all young people or professionals.

Although we were able to discuss some findings from the gap analysis with available self-harm and suicide literature around guidelines for schools [19, 21], there is an overall lack of empirical literature around evidence-based guidelines for UK schools and youth organisations, therefore we were unable to consider our findings in the context of evidence of what is effective in a UK context. There remains a need to understand the most helpful features to effectively support education and youth settings from the perspectives of the people in those settings (both young people and professionals). Future research would benefit from conducting qualitative work to gain a better understanding of what young people and staff in these settings need, what they feel currently works well and what they feel is missing from current guidance identified within this review. Further, there is a need to test the effectiveness of guidance by seeing if these guidance resources improve outcomes in real-life scenarios.

Another limitation is that we did not look at the physical format of guidance resources such as length, complexity of language - factors that might influence whether they are read and implemented.

## Conclusion

This review is the first to provide an overview of publicly available prevention and postvention guidance on self-harm and suicide for education and youth settings in the UK. Both a content analysis and a gap analysis of key features of 104 included guidance documents was conducted. Key findings revealed a lack of clarity around the evidence-base of current guidance and therefore difficulty assessing the quality of existing guidance. The key gaps identified were a lack of guidance for youth organisations, a lack of postvention guidance (support after a death by suicide), a lack of tailored information for specific populations (e.g. LGBTQIA+, neurodiverse young people), information for how organisations can support parents, carers and families and practical advice to address issues relating to the internet and social media as well as a lack of practical resources for professionals to use (e.g. templates and checklists). Taken together, the findings point to an urgent need for guidance that is co-produced with young people and professionals, clearly evidence-informed, and provides clear, practical recommendations.

These findings have clear implications for policy, training, and implementation. This review supports the development of coordinated, evidence-informed national guidance that is applicable across education and wider youth settings, with a stronger emphasis on postvention and whole-system responses. In policy terms, there is a need for greater alignment between national suicide prevention strategies and the practical guidance available to frontline organisations, ensuring that both are underpinned by evidence-informed strategies.

There is also a need for strengthened training provision and ongoing professional support to build confidence, competence, and consistency in responding to self-harm and suicide, particularly in non-education youth settings where access to structured guidance may be more limited. Training frameworks should extend beyond awareness-raising to include skills-based and scenario-based learning. In addition, training should be accompanied by clear implementation guidance and practical toolkits, such as templates, flowcharts, and example protocols, to support translation into routine practice.

Finally, there is a clear need for a dedicated UK-based Delphi study to establish expert and stakeholder consensus on priority content and delivery of future guidance, addressing a current gap in the UK literature and informing policy-aligned, practice-ready guidance for those working with children and young people.

## Supplementary Information

Supplementary Material 1.

## Data Availability

All data generated or analysed within this review are included in this published article and its supplementary information files.

## Abbreviations

ASIST: Applied Suicide Intervention Skills Training
CAMHS: Child and Adolescent Mental Health Services
LGBTQIA+: lesbian, gay, bisexual, transgender, queer or questioning, intersex, and asexual
NHS: National Health Service
NICE: National Institute for Health and Care Excellence
NSPCC: The National Society for the Prevention of Cruelty to Children
PRISMA: Preferred Reporting Items for Systematic reviews and Meta-Analyses
PSHE: Personal, Social, Health, and Economic education
SEND: Special Educational Needs and Disabilities
SH&S: Self-harm and Suicide
VCSE: Voluntary, Community and Social Enterprise
YPAG: Young Person’s Advisory Group
