# Supplementary material for: Prevention and postvention guidance relating to self-harm and suicide for UK educational and youth organisations: a systematic review of grey literature
Source: BMC Public Health. 2026 Mar 18;26:1376. doi: 10.1186/s12889-026-27052-6 (PMC13112647; doi:10.1186/s12889-026-27052-6)
Supplement: Supplementary file 1 — Supplementary Material 1. [file 12889_2026_27052_MOESM1_ESM.pdf]

Supplementary Materials

Supplementary Materials 1: PRISMA Checklist

Section and Topic	Item #	Checklist item	Location where item is reported
**TITLE**			
Title	1	Identify the report as a systematic review.	Page 1
**ABSTRACT**			
Abstract	2	See the PRISMA 2020 for Abstracts checklist.	Page 2
**INTRODUCTION**			
Rationale	3	Describe the rationale for the review in the context of existing knowledge.	Pages 3-4
Objectives	4	Provide an explicit statement of the objective(s) or question(s) the review addresses.	Page 4
**METHODS**			
Eligibility criteria	5	Specify the inclusion and exclusion criteria for the review and how studies were grouped for the syntheses.	Page 5
Information sources	6	Specify all databases, registers, websites, organisations, reference lists and other sources searched or consulted to identify studies. Specify the date when each source was last searched or consulted.	Pages 4-5
Search strategy	7	Present the full search strategies for all databases, registers and websites, including any filters and limits used.	Page 4 (link to review protocol)
Selection process	8	Specify the methods used to decide whether a study met the inclusion criteria of the review, including how many reviewers screened each record and each report retrieved, whether they worked independently, and if applicable, details of automation tools used in the process.	Pages 5-6
Data collection process	9	Specify the methods used to collect data from reports, including how many reviewers collected data from each report, whether they worked independently, any processes for obtaining or confirming data from study investigators, and if applicable, details of automation tools used in the process.	Page 6
Data items	10a	List and define all outcomes for which data were sought. Specify whether all results that were compatible with each outcome domain in each study were sought (e.g. for all measures, time points, analyses), and if not, the methods used to decide which results to collect.	Page 6
	10b	List and define all other variables for which data were sought (e.g. participant and intervention characteristics, funding sources). Describe any assumptions made about any missing or unclear information.	Page 6
Study risk of bias assessment	11	Specify the methods used to assess risk of bias in the included studies, including details of the tool(s) used, how many reviewers assessed each study and whether they worked independently, and if applicable, details of automation tools used in the process.	N/A
Effect measures	12	Specify for each outcome the effect measure(s) (e.g. risk ratio, mean difference) used in the synthesis or presentation of results.	N/A
Synthesis methods	13a	Describe the processes used to decide which studies were eligible for each synthesis (e.g. tabulating the study intervention characteristics and comparing against the planned groups for each synthesis (item #5)).	N/A
	13b	Describe any methods required to prepare the data for presentation or synthesis, such as handling of missing summary statistics, or data conversions.	N/A
	13c	Describe any methods used to tabulate or visually display results of individual studies and syntheses.	N/A
	13d	Describe any methods used to synthesize results and provide a rationale for the choice(s). If meta-analysis was performed, describe the model(s), method(s) to identify the presence and extent of statistical heterogeneity, and software package(s) used.	N/A
	13e	Describe any methods used to explore possible causes of heterogeneity among study results (e.g. subgroup analysis, meta-regression).	N/A
	13f	Describe any sensitivity analyses conducted to assess robustness of the synthesized results.	N/A
Reporting bias assessment	14	Describe any methods used to assess risk of bias due to missing results in a synthesis (arising from reporting biases).	N/A
Certainty	15	Describe any methods used to assess certainty (or confidence) in the body	N/A

Section and Topic	Item #	Checklist item	Location where item is reported
assessment		of evidence for an outcome.	
**RESULTS**			
Study selection	16a	Describe the results of the search and selection process, from the number of records identified in the search to the number of studies included in the review, ideally using a flow diagram.	Page 7
	16b	Cite studies that might appear to meet the inclusion criteria, but which were excluded, and explain why they were excluded.	N/A
Study characteristics	17	Cite each included study and present its characteristics.	Supplementary materials 1
Risk of bias in studies	18	Present assessments of risk of bias for each included study.	N/A
Results of individual studies	19	For all outcomes, present, for each study: (a) summary statistics for each group (where appropriate) and (b) an effect estimate and its precision (e.g. confidence/credible interval), ideally using structured tables or plots.	N/A
Results of syntheses	20a	For each synthesis, briefly summarise the characteristics and risk of bias among contributing studies.	N/A
	20b	Present results of all statistical syntheses conducted. If meta-analysis was done, present for each the summary estimate and its precision (e.g. confidence/credible interval) and measures of statistical heterogeneity. If comparing groups, describe the direction of the effect.	Pages 10-18
	20c	Present results of all investigations of possible causes of heterogeneity among study results.	N/A
	20d	Present results of all sensitivity analyses conducted to assess the robustness of the synthesized results.	N/A
Reporting biases	21	Present assessments of risk of bias due to missing results (arising from reporting biases) for each synthesis assessed.	N/A
Certainty of evidence	22	Present assessments of certainty (or confidence) in the body of evidence for each outcome assessed.	N/A
**DISCUSSION**			
Discussion	23a	Provide a general interpretation of the results in the context of other evidence.	Pages 19-22
	23b	Discuss any limitations of the evidence included in the review.	Page 22
	23c	Discuss any limitations of the review processes used.	Page 22
	23d	Discuss implications of the results for practice, policy, and future research.	Page 22-23
**OTHER INFORMATION**			
Registration and protocol	24a	Provide registration information for the review, including register name and registration number, or state that the review was not registered.	Page 4
	24b	Indicate where the review protocol can be accessed, or state that a protocol was not prepared.	Page 4
	24c	Describe and explain any amendments to information provided at registration or in the protocol.	N/A
Support	25	Describe sources of financial or non-financial support for the review, and the role of the funders or sponsors in the review.	Page 23
Competing interests	26	Declare any competing interests of review authors.	None to declare
Availability of data, code and other materials	27	Report which of the following are publicly available and where they can be found: template data collection forms; data extracted from included studies; data used for all analyses; analytic code; any other materials used in the review.	On journal submission

From: Page MJ, McKenzie JE, Bossuyt PM, Boutron I, Hoffmann TC, Mulrow CD, et al. The PRISMA 2020 statement: an updated guideline for reporting systematic reviews. BMJ 2021;372:n71. doi: 10.1136/bmj.n71. This work is licensed under CC BY 4.0. To view a copy of this license, visit https://creativecommons.org/licenses/by/4.0/

Supplementary Materials 2: Google Search Terms

<u>Advanced Google Searches:</u>

<u>Search 1</u>

Self-harm OR self-injury OR suicide OR suicidal OR critical incident ***and***

Guidance OR guidelines OR guide OR prevention OR intervention OR support OR management OR protocol OR policy OR procedure OR toolkit OR recommendations OR safeguarding ***and***

Any of these words:

Child OR children OR young person OR young people OR youth OR adolescent OR adolescents OR adolescence OR 11-18 years OR teen OR teenager OR teenagers OR school OR college OR education OR local authority OR youth club OR youth organisation youth group OR sport OR sports OR sport club OR football OR rugby OR basketball OR netball hockey OR gymnastics OR dancing OR golf OR cricket tennis table tennis OR athletics OR cycling OR chess OR photography OR martial arts OR swimming OR camping OR climbing OR hiking OR surfing OR sailing OR kayaking OR scouts OR guides OR sea cadets OR choir OR orchestra OR music OR religious group OR political group OR grassroots OR support OR guidance OR guidelines OR policy OR protocol OR advice OR resource OR toolkit OR recommendations

<u>Search 2</u>

Suicide or critical incident **and** postvention OR guidance OR guidelines OR guide OR prevention OR intervention OR support OR management OR protocol OR policy OR procedure toolkit OR recommendations OR safeguarding **and**

Any of these words:

Child OR children OR young people OR youth OR adolescent OR adolescents OR adolescence OR 11-18 years OR teen OR teenager OR teenagers OR school OR college OR education OR local authority OR youth club OR youth organisation youth group OR sport OR sports OR sport club OR football OR rugby OR basketball OR netball hockey OR gymnastics OR dancing OR golf OR cricket tennis table tennis OR athletics OR cycling OR chess OR photography OR martial arts OR swimming OR camping OR climbing OR hiking OR surfing OR sailing OR kayaking OR scouts OR guides OR sea cadets OR choir OR orchestra OR music OR religious group OR political group OR grassroots OR support OR guidance OR guidelines OR policy OR advice OR resource OR toolkit OR recommendations

Supplementary Materials 3: Full Table of Included Guidance Documents, Guidance Type and Key Features

	Guidance Type					Psychoeducation/Information						Recommended Response									Resources				Additional considerations	
Guidance	Self-harm	Suicide	Prevention	Postvention	Critical incident/unexpected death/bereavement	Definitions/Information	Prevalence/Incidence	Risk factors/triggers	Myths/Misconceptions	Internet/social media	Confidentiality/Consent	Conversations and language	Coping strategies/self-care	Contagion/clustering	Risk assessment	General well-being promotion in schools	Signposting	Handling the media (death by suicide only)	Legal issues	Funerals/memorials (Postvention specific)	Templates/resources (safety plan, checklists & incident forms)	Framework/Diagram	Information for parents/carers	Support/training for staff	Tailored guidance for specific populations/vulnerable groups (culture, religious, LGBT, SEN)	Information on guidance development
A guide to help schools prepare and respond to a critical incident		✓		✓	✓	✓				✓					✓		✓								✓	
A Guide to Managing Critical Incidents In Schools		✓		✓	✓	✓				✓	✓				✓		✓	✓								
A Whole School Approach to Self Harm Awareness and Training A Knowledge and Skills Framework	✓		✓			✓	✓	✓					✓			✓	✓					✓	✓	✓		✓
Aberdeen City Multiagency Guidance for people working with children and young people at risk of self harm, April 2018	✓		✓			✓		✓			✓		✓		✓		✓		✓							✓
Advice for sports organisations when addressing self-harming behaviour	✓		✓			✓	✓	✓					✓		✓		✓									
Advice for sports organisations when addressing suicidal thoughts and feelings		✓	✓					✓				✓	✓				✓									
Advice to school leaders following a suspected suicide		✓		✓		✓				✓	✓	✓		✓			✓	✓								
Ayrshire multi-agency guidance for people working with children and young people at risk of self-harm or suicide September 2013	✓	✓	✓	✓		✓	✓	✓			✓	✓	✓		✓		✓									✓
Building Suicide-Safer Schools and Colleges, A guide for teachers and staff		✓	✓	✓		✓	✓	✓		✓		✓	✓		✓		✓									
Cheshire and Merseyside Children and Young Persons Self-Harm Practice Guide, November 2021	✓		✓			✓	✓	✓			✓	✓	✓		✓		✓									✓
Choose Life Joint Multi-Agency Guidance Children and young people at risk of self harm and suicide	✓	✓	✓			✓	✓	✓				✓	✓		✓		✓									✓
Coping After Suicide or Sudden Traumatic Death: A guide for schools		✓		✓		✓		✓		✓		✓	✓		✓		✓	✓								✓
Coping with Suicide A summary of support for educational settings 2021		✓		✓														✓		✓						
Critical incident guidance for schools and other educational settings		✓		✓	✓	✓	✓	✓				✓	✓	✓	✓		✓	✓	✓							
Critical incident pack – information for schools and educational settings		✓		✓	✓	✓		✓				✓	✓		✓		✓	✓								
Critical Incident Response Pack, Plymouth Educational Psychology Service		✓		✓	✓	✓		✓				✓	✓		✓		✓	✓		✓						
Critical Incidents in Educational Communities Guidance on creating the best environment for recovery		✓		✓	✓			✓		✓	✓	✓	✓		✓		✓	✓		✓				✓	✓	✓
East Dunbartonshire inter-agency guidance for educational establishments working with young people at risk of self-harm or suicide	✓	✓	✓			✓	✓	✓			✓	✓	✓	✓	✓	✓	✓		✓							
Girlguiding: self-harm, how to support members who self-harm	✓		✓			✓	✓	✓			✓	✓	✓		✓		✓									
Guidance for Educational Settings Following a Suicide or Sudden Death		✓		✓		✓	✓	✓	✓	✓		✓		✓			✓		✓	✓	✓	✓				
Guidance for Somerset Schools and Colleges in Managing Critical Incidents		✓		✓	✓	✓	✓	✓		✓		✓	✓	✓	✓		✓	✓	✓	✓	✓	✓	✓	✓		

**Guidelines and procedure to support young people who are self-harming or engaging in suicide behaviours in Lanarkshire**	✓	✓	✓			✓	✓	✓	✓			✓	✓	✓	✓		✓				✓					
**Guidelines and resources for schools to help support children and young people who self-harm**	✓		✓			✓	✓	✓	✓			✓	✓	✓	✓		✓									
**Help when we needed it most, how to prepare for and respond to suicide in schools and colleges**		✓		✓		✓	✓	✓			✓	✓	✓		✓		✓			✓						
**Hertfordshire Suicide Aware Prevention Intervention and Postvention Charter**		✓	✓	✓		✓		✓			✓	✓	✓	✓			✓						✓	✓		
**How to prepare for and respond to a suspected suicide in schools, colleges and other youth settings in the UK, Postvention**		✓		✓				✓		✓		✓		✓			✓	✓		✓						
**How to Respond to Self-Harm and Suicide Ideation**	✓	✓	✓					✓	✓			✓				✓	✓						✓			
**Inter Agency Guidance for those working with Children and Young People Involved in Self-harm and Suicide 2nd Edition February 2013**	✓	✓	✓	✓		✓	✓	✓	✓		✓	✓			✓		✓					✓	✓	✓		
**Let's Talk: Reducing the Risk of Suicide Promoting positive emotional wellbeing and reducing the risk of suicidal thoughts and actions in Children and Young People, Guidance for Educational Settings February 2021**		✓	✓	✓						✓																
**Liverpool Multi-Agency Self-Harm Practice Guidance**	✓		✓			✓	✓	✓	✓		✓		✓				✓				✓	✓				
**LSCP management of young people who self-harm or have suicidal ideation for all staff and volunteers working with children and young people in Sutton**	✓	✓	✓			✓	✓	✓		✓					✓							✓	✓		✓	
**Managing bereavement: A guide for schools**		✓		✓	✓					✓										✓					✓	
**Managing self-harm Practical guidance and toolkit for schools in Cornwall and the Isles of Scilly**	✓		✓			✓	✓	✓			✓	✓		✓	✓											
**Managing self-harm Practical guidance for schools November 2014**	✓		✓			✓		✓	✓	✓	✓	✓	✓		✓		✓				✓	✓	✓			
**Managing the Response to Critical Incidents in Schools, Settings and Services**		✓		✓		✓		✓		✓	✓	✓			✓		✓			✓	✓	✓		✓		
**Milton Keynes Self-Harm Toolkit: Guidelines and resources to help support children and young people who self-harm School Edition.**	✓		✓			✓			✓				✓				✓				✓	✓	✓	✓	✓	
**Model guidance: Schools responding to incidents of self-harm**	✓		✓			✓	✓	✓	✓		✓	✓		✓	✓		✓				✓		✓	✓		
**Moray Multi-agency Guidance for people working with children and young people at risk of self-harm**	✓		✓			✓					✓						✓		✓		✓	✓	✓	✓		
**Multi-Agency Guidance for Staff in Universal Services Working with Young People Who Self-Harm 2nd Edition April 2019**	✓		✓			✓	✓	✓	✓		✓	✓	✓	✓	✓		✓				✓	✓	✓	✓		
**Multi-agency guidance for staff working with young people at risk of self-harm and/or suicide**	✓	✓	✓	✓		✓	✓	✓		✓	✓	✓	✓		✓		✓		✓		✓	✓	✓	✓	✓	
**North Yorkshire Pathway of support for children and young people with self harming behaviour and/or suicidal ideation**	✓	✓	✓			✓	✓	✓	✓	✓	✓	✓	✓	✓	✓	✓	✓		✓		✓	✓	✓	✓	✓	
**Post Suicide Intervention Protocol following an unexpected death by suspected suicide of a child or young person**		✓		✓		✓	✓	✓		✓	✓	✓		✓	✓	✓	✓	✓		✓		✓	✓	✓		
**Preparing for and Responding to a Student Death by Suicide, A Buckinghamshire Suicide Prevention and Postvention Guide for Schools and Colleges**		✓	✓	✓		✓	✓	✓	✓	✓	✓	✓	✓	✓	✓	✓	✓	✓			✓	✓	✓	✓		
**Prevention and Postvention Protocol for Isle of Wight Schools and Colleges**		✓	✓	✓			✓			✓		✓				✓	✓	✓		✓				✓		✓
**Procedure for the Management of SelfHarm and or Suicidal Behaviour in Children & Young People**	✓	✓	✓			✓		✓	✓		✓	✓			✓		✓		✓			✓	✓		✓	
**Professional Resource Pack for Supporting Young People with Self-harm & Suicidal Behaviours**	✓	✓	✓			✓		✓		✓		✓	✓		✓	✓	✓				✓	✓				
**Protecting children in the City of York, Self-harm and suicidal behaviour Working with children and young people in York**	✓	✓	✓			✓	✓	✓				✓		✓	✓		✓						✓	✓	✓	
**Protecting life in schools, Helping Protect Against Suicide by Supporting Pupils' Emotional Health and Wellbeing**		✓	✓			✓	✓	✓	✓	✓	✓	✓				✓	✓				✓	✓		✓		
**Protocol for the Management of Self-harm in the Community**	✓	✓	✓			✓	✓	✓	✓			✓			✓	✓	✓					✓	✓			
**Responding in the event of a young person's suspected suicide**		✓		✓				✓	✓	✓		✓					✓					✓	✓	✓		
**Responding in the event of a young person's suspected suicide.**		✓		✓		✓		✓	✓	✓		✓	✓		✓		✓	✓					✓	✓		
**Responding to a potential cluster of suicides for children and young people aged under 18**		✓		✓		✓		✓		✓	✓			✓	✓		✓	✓					✓	✓		
**Responding to an Unexpected Death: Support for Schools & Colleges**		✓		✓	✓					✓		✓	✓	✓		✓		✓	✓				✓	✓	✓	
**Responding to Critical Incidents (Schools) - Information Pack**		✓		✓	✓	✓		✓				✓						✓			✓					

**Responding to Critical Incidents A Guide and Resources for Schools and Settings**		✓		✓	✓				✓	✓		✓					✓	✓			✓	✓				
**Responding to Critical Incidents Resource Materials for Schools**		✓		✓	✓			✓	✓	✓	✓	✓	✓	✓	✓	✓	✓	✓	✓				✓	✓		
**Responding to issues of self-harm and thoughts of suicide in young people, guidance for teachers, professionals, volunteers and youth services**	✓	✓	✓			✓	✓	✓		✓	✓		✓		✓		✓				✓	✓				
**Response to a suspected suicide**		✓		✓						✓				✓	✓			✓					✓			
**Rotherham Self-Harm Practice Guidance**	✓		✓			✓	✓	✓	✓	✓	✓	✓	✓	✓	✓	✓	✓		✓		✓	✓	✓	✓		
**Scottish Borders Guidance: a multi-agency approach to working with young people at risk of self-harm and suicide**	✓	✓	✓			✓		✓				✓			✓		✓		✓					✓		
**Self Harm and Suicidal Behaviour in Children and Young People**	✓	✓	✓			✓		✓		✓	✓		✓		✓		✓				✓	✓	✓			
**Self harm Guidelines for staff within school and residential settings in Oxfordshire**	✓		✓			✓		✓		✓	✓	✓	✓		✓		✓		✓		✓	✓	✓			✓
**Self Harm Information and suggestions for school staff**	✓		✓			✓	✓	✓			✓		✓				✓				✓	✓		✓		
**Self-harm A CAMHS designed management tool kit of education and community settings**	✓		✓			✓		✓			✓	✓				✓	✓				✓		✓			
**Self-harm and Health-Harming Behaviours Guidance for Schools and Settings**	✓		✓			✓		✓				✓					✓						✓	✓		
**Self-harm and Suicidal Behaviour**	✓	✓	✓			✓		✓		✓	✓	✓			✓		✓									
**Self-harm and Suicidal Behaviour Guidance Working with children and young people in Derby City and Derbyshire September 2020**	✓	✓	✓			✓		✓		✓	✓		✓		✓		✓				✓					
**Self-Harm Guidance for Schools in Bristol, South Gloucestershire and North Somerset**	✓		✓			✓	✓	✓		✓	✓	✓	✓	✓	✓	✓	✓		✓		✓		✓	✓		
**Self-Harm Guidelines for School Staff**	✓		✓			✓	✓	✓					✓		✓		✓				✓		✓			✓
**Self-harm Information for school and college staff in Hertfordshire**	✓	✓	✓			✓		✓	✓	✓	✓	✓	✓		✓	✓					✓	✓	✓	✓		
**Self-Harm Pathway Information, Advice and Guidance for Practitioners**	✓		✓			✓	✓	✓			✓	✓	✓	✓	✓		✓				✓		✓			
**Self-harm Policy: Secondary Schools**	✓	✓	✓			✓	✓	✓			✓	✓	✓	✓	✓	✓	✓					✓	✓	✓		
**Self-Harm Toolkit Guidelines and resources for East Sussex schools and colleges to help support students who self-harm or are at risk of engaging in self-harming behaviour**	✓		✓			✓	✓	✓		✓	✓	✓	✓		✓	✓	✓							✓	✓	
**Self-Harm, Guidance for School Based Staff**	✓		✓			✓		✓			✓						✓									
**Self-harm, suicidal behaviour & suicide**	✓	✓	✓	✓		✓		✓		✓	✓			✓	✓		✓						✓			
**Self-harm: Guidance**	✓		✓			✓	✓	✓			✓		✓	✓							✓	✓	✓	✓		
**Staffordshire Safeguarding Children Board (SSCB) Section 4U Children and Young People who Self Harm or Disclose Intent to Die by Suicide**	✓	✓	✓			✓	✓	✓	✓	✓	✓						✓				✓	✓				
**Sudden and Traumatic Death and Bereavement A Guide for Schools and Educational Settings in Cumbria**		✓		✓	✓	✓	✓	✓		✓	✓	✓	✓	✓	✓	✓	✓	✓			✓		✓	✓		
**Suicide and self harm - Barnet - WWC**	✓	✓	✓	✓		✓	✓	✓	✓		✓	✓			✓	✓	✓							✓		
**Suicide Postvention Guidance for Schools: Responding to a pupil suicide**		✓		✓		✓				✓				✓				✓		✓	✓		✓	✓		✓
**Suicide Prevention & Postvention Protocol Guidance for Schools and Colleges in Hampshire**		✓	✓	✓		✓	✓	✓		✓	✓	✓		✓	✓	✓	✓	✓						✓		
**Suicide Prevention and Postvention Protocol for all Surrey Schools and Colleges**		✓	✓	✓		✓		✓		✓		✓				✓	✓	✓						✓		
**Suicide Prevention and Postvention Protocol for Portsmouth Schools and Colleges**		✓	✓	✓		✓	✓	✓		✓		✓	✓	✓			✓	✓				✓	✓	✓		
**Suicide Prevention Pathway for Children and Young People in Sheffield March 2017**		✓	✓			✓	✓	✓				✓			✓		✓	✓				✓		✓		
**Support after Suicide Partnership, Talking to Students**		✓		✓		✓						✓					✓									
**Supporting Children & Young People who Self Harm: Rotherham Self Harm Practice Guidance**	✓		✓			✓	✓	✓			✓	✓	✓	✓	✓	✓	✓				✓	✓	✓	✓		
**Supporting Children and Young People at Risk of Self Harm and Suicide Tayside Multi-Agency Guidance**	✓	✓	✓	✓		✓	✓	✓	✓		✓	✓			✓	✓	✓						✓	✓	✓	

**Supporting Children and Young People at Risk of Self Harm West Lothian Multi-Agency Guidance**	✓		✓			✓		✓	✓								✓				✓		✓	✓		
**Supporting Children and Young People Presenting with Self-Harming Behaviours, or Intent to Self-Harm**	✓		✓			✓	✓	✓			✓			✓			✓				✓	✓				✓
**Supporting Children and Young People who Self-Harm Guidelines for those working with children and young people**	✓		✓			✓	✓	✓			✓		✓	✓			✓				✓	✓				
**Supporting children and young people who self-harm: guidelines for school staff**	✓		✓			✓	✓	✓			✓	✓	✓	✓			✓				✓	✓				
**Supporting children bereaved by suicide**		✓		✓		✓						✓														
**Supporting Schools and Colleges in responding to suicides in teenagers: A multi-agency guide for practitioners.**		✓		✓						✓				✓	✓		✓					✓		✓		
**Supporting young people who self-harm or feel suicidal A guide for professionals in Leeds working with young people aged 25 and under**	✓	✓	✓			✓		✓		✓	✓	✓	✓								✓	✓			✓	
**Surrey Safeguarding Children Partnership Self-Harm Protocol Multi-Agency Practice Guidance for Dealing with Incidents of Self-Harm**	✓		✓			✓	✓	✓		✓	✓	✓	✓		✓	✓						✓		✓		
**Talking to Young People About Suicide**		✓	✓				✓					✓					✓						✓	✓		
**Tees Safeguarding Children Partnerships' Procedures: Deliberate Self-harm**	✓		✓			✓		✓			✓				✓		✓					✓		✓		
**The Boys Brigade, Self-harming: Be Aware**	✓		✓			✓		✓									✓					✓				
**Understanding and responding to children and young people at risk of self-harm and suicide**	✓	✓	✓			✓		✓	✓	✓	✓	✓			✓		✓					✓		✓	✓	
**Understanding and Responding to Children and Young People at risk of Self-Harm and Suicide A guide for adults who work with young people in Fife**	✓	✓	✓			✓	✓	✓	✓	✓	✓	✓			✓		✓		✓		✓	✓	✓	✓	✓	
**Wavepower 2020-2023, Swim England Child Safeguarding Policies and Procedures**	✓		✓			✓	✓	✓		✓		✓					✓									
**Young People & Self-Harm in Stockport Policy & Procedures: Schools' Version**	✓		✓			✓	✓	✓				✓	✓			✓					✓	✓	✓	✓		
**Young People and Self-Harm : Guidance for Schools**	✓		✓			✓	✓	✓			✓	✓	✓	✓			✓				✓	✓	✓			
**Young people who self-harm A Guide for School Staff**	✓		✓			✓	✓			✓	✓	✓		✓			✓									

Supplementary Materials 4: Example Letter to Parents

Appendix B

Sample letter to parents following meeting about self harm

Dear (Parent/Carer)

Thank you for coming to discuss.......................................

After our recent meeting I am writing to express concern about ....'s safety and welfare. The recent incident of self harm (or threat to self harm) by .... suggests that he/she may need professional help.

I recommend that you see your GP for advice and help and / or as agreed, we have sent a referral to PCAMHS.

We will provide support to ...., including referral to school counselling if indicated, but would appreciate any information that you feel would help us to do this as effectively as possible.

If there is anything else we can do to help .... please contact me.

Yours sincerely,

Title

Copies to:

Oxfordshire Adolescent Self-harm Forum (2016) Self harm Guidelines for staff within school and residential settings in Oxfordshire

Supplementary Materials 5: Vulnerable Students Log Example

Vulnerable students log

Name	Year/Form	Context	Current support	Lead staff member	Check-in record
Joe Bloggs	10R	– Father ended his life last year.<br>– Regular self-harm.<br>– In the same English set as the deceased.	– Has bereavement therapy outside of school.<br>– Year 12 academic mentor.<br>– Time-out card to use in case of emergency.	Miss Jones – Form Tutor	09/06 – Miss Jones had 1-2-1 with Joe. He has mixed emotions at the moment but seems to be managing things well.<br>12/06 – Mrs White contacted Joe's mother to update on Joe and check his wellbeing at home. Agreed that he seems to be coping and really benefits from his year 12 academic mentor. Has not used his time-out this week.

Supplementary Materials 6: Examples of frameworks and diagrams

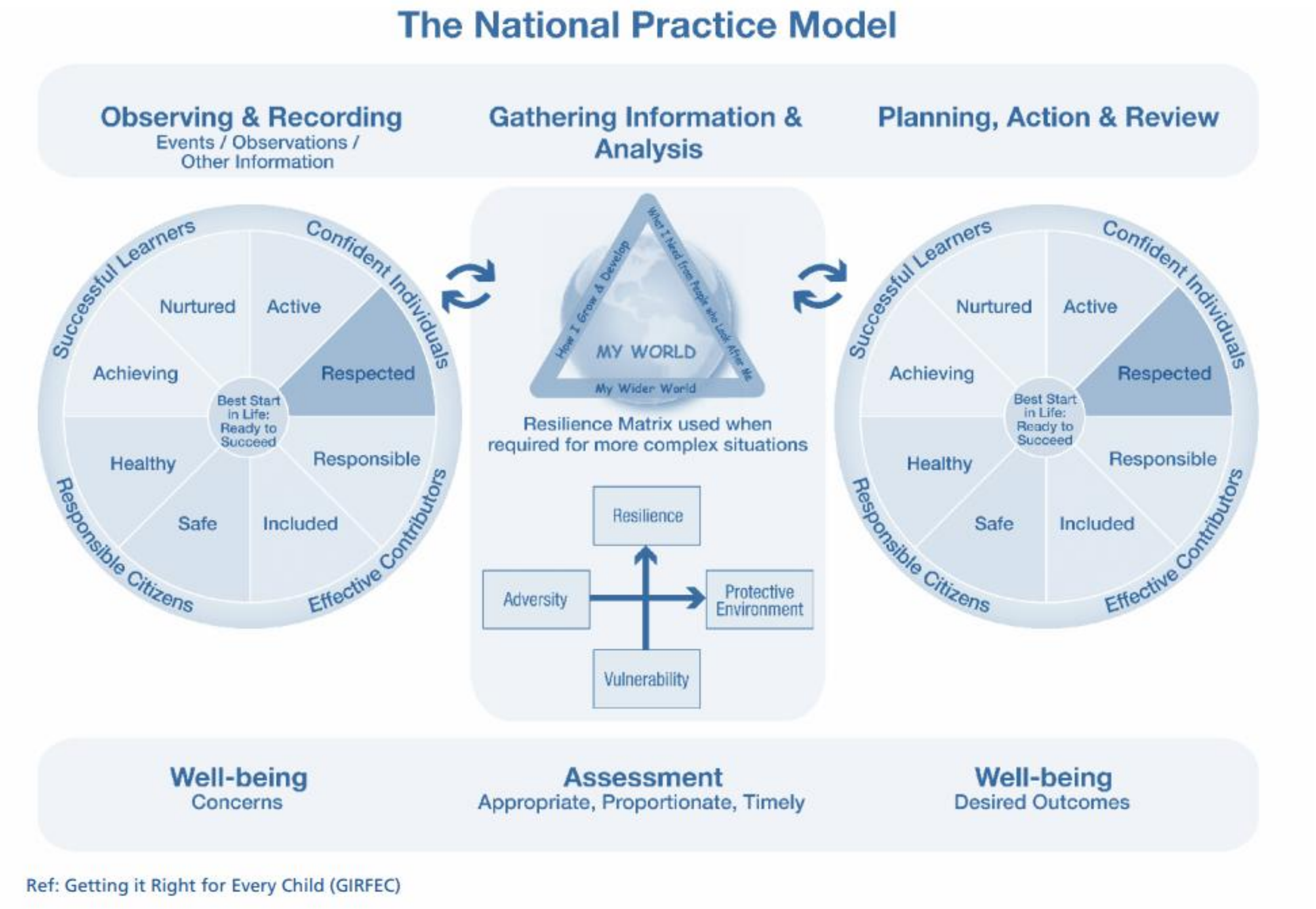

West Dunbartonshire, Inter Agency Guidance for those working with Children and Young People Involved in Self-harm and Suicide, 2nd Edition February 2013

The cycle of self harm/cutting

When a person inflicts pain upon himself or herself the body responds by producing endorphins, a natural pain reliever that gives temporary relief or a feeling of peace. The addictive nature of this feeling can make self harm difficult to stop.

Young people who self harm still feel pain, but some say the physical pain is easier to stand than the emotional/mental pain that initially led to the self harm.

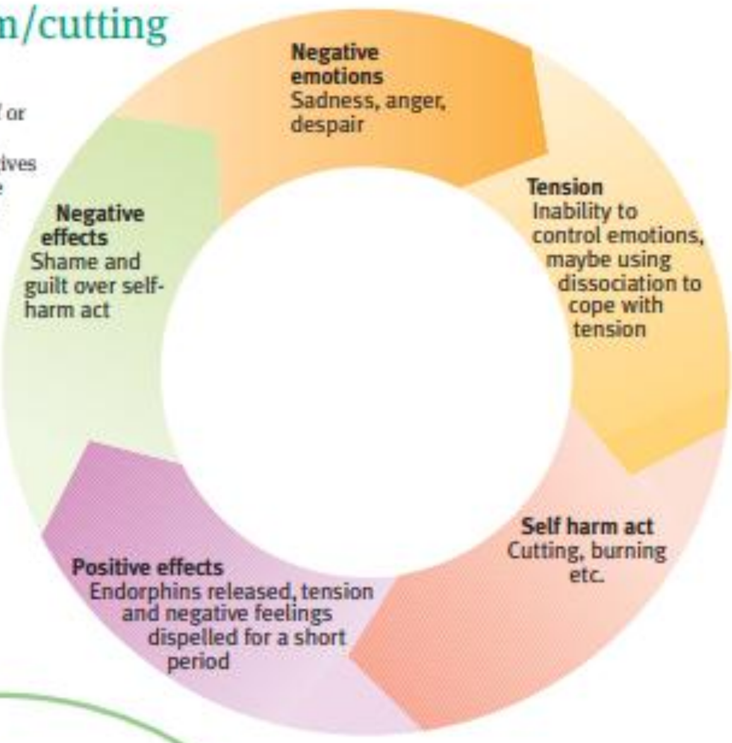

Coping strategies

1 Using support networks

It is helpful to identify who can support the young person and how to get in touch with them. Examples are friends, family, school teacher, counsellor. Knowing how to access a crisis line is also important. **"My safety net"** (see appendix A) can be a useful way of recording the support people in a young person's life.

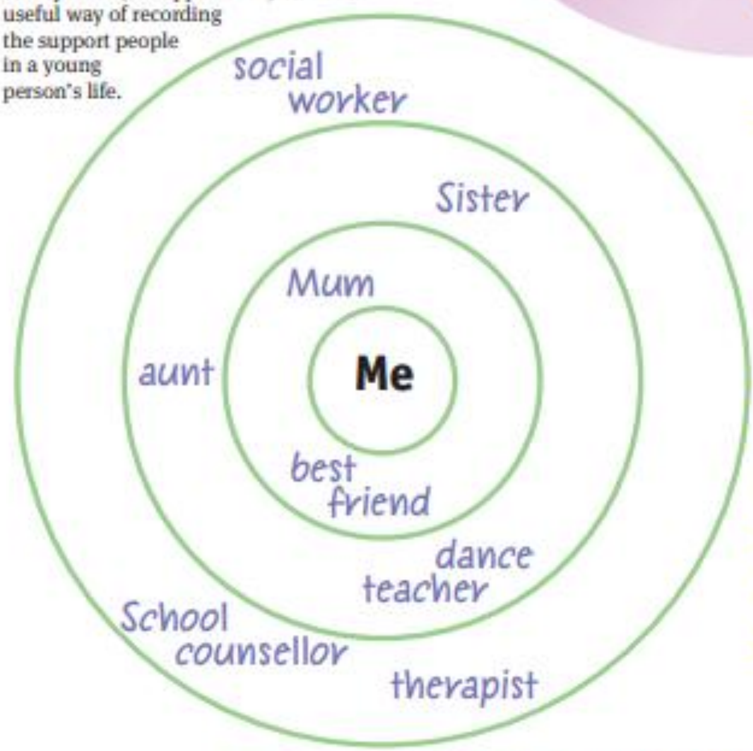

2 Distraction activities

Replacing the cutting or other self harm with other safer activities can be a positive way of coping with the tension. What works depends on the reasons behind the self harm. Activities that involve the emotions intensely can be helpful.

Examples of distraction methods:

- Contacting a friend or family member
- Going for a walk/run or other forms of physical exercise
- Getting out of the house and going to a public place e.g. a cinema
- Reading a book
- Keeping a diary
- Looking after an animal
- Watching TV
- Listening to music

Things I can do myself to cope with difficult feelings:

- Ring my friend up
- Have a relaxing bath
- Watch a film on TV
- Go for a walk
- Write down my feelings
- Listen to my favourite music

Oxfordshire Adolescent Self-harm Forum (2016) Self harm Guidelines for staff within school and residential settings in Oxfordshire

Supplementary Materials 7: Examples of information sheet tailored to young people

Appendix F Information sheet for young people on self harm

What is self harm?

Self harm is when someone does something to deliberately hurt him or herself. This may include: cutting parts of their body, burning, hitting or taking an overdose.

How many young people self harm?

A recent large study in the UK found that about 7% (i.e. 7 people out of every 100) of 15-16 year olds had self harmed in the last year.

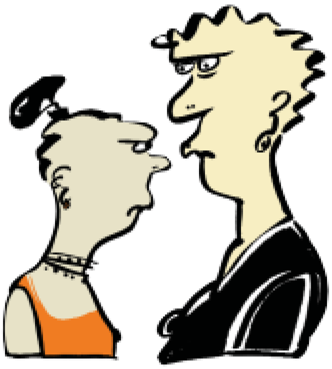

Why do young people self harm?

Self harm is often a way of trying to cope with painful and confusing feelings.

Difficult things that people who self harm talk about include:

- Feeling sad or feeling worried
- Not feeling very good or confident about themselves
- Being hurt by others: physically, sexually or emotionally
- Losing someone close; this could include someone dying or leaving
- Feeling under a lot of pressure at school or at home

When difficult or stressful things happen in someone's life, it can trigger self harm.

Upsetting events that might lead to self harm include:

- Arguments with family or friends
- Break-up of a relationship
- Failing (or thinking you are going to fail) exams
- Being bullied

Often these things build up until the young person feels they cannot cope anymore. Self harm can be a way of trying to deal with or escaping from these difficult feelings. It can also be a way of showing other people that something is wrong in their lives.

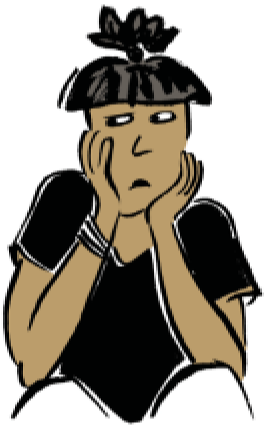

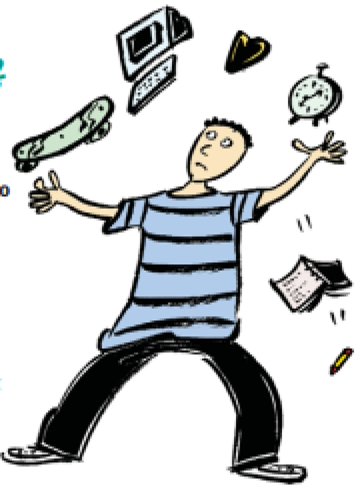

How can you cope with self harm?

Replacing the self harm with other safer coping strategies can be a positive and more helpful way of dealing with difficult things in your life.

Helpful strategies can include:

- Finding someone to talk to about your feelings (this could be a friend or family member)
- Talking to someone on the phone (you might want to ring a help line)
- Sometimes it can be hard to talk about feelings; writing and drawing about your feelings may help.
- Scribbling on and/or ripping up paper
- Listening to music
- Going for a walk, run or other kinds of exercise
- Getting out of the house and going somewhere where there are other people
- Keeping a diary
- Having a bath/using relaxing oils e.g. lavender
- Hitting a pillow or other soft object
- Watching a favourite film

Oxfordshire Adolescent Self-harm Forum (2016) Self harm Guidelines for staff within school and residential settings in Oxfordshire
